# Phage-antibiotic synergy: Cell filamentation is a key driver of successful phage predation

**DOI:** 10.1371/journal.ppat.1011602

**Published:** 2023-09-13

**Authors:** Julián Bulssico, Irina PapukashvilI, Leon Espinosa, Sylvain Gandon, Mireille Ansaldi

**Affiliations:** 1 Laboratoire de Chimie Bactérienne, UMR7283, Centre National de la Recherche Scientifique, Aix-Marseille Université, Marseille, France; 2 Faculty of Exact and Natural Sciences, Ivane Javakhishvili Tbilisi State University, Tbilisi, Georgia; 3 CEFE, CNRS, Univ Montpellier, EPHE, IRD, Montpellier, France; Harvard Medical School, UNITED STATES

## Abstract

Phages are promising tools to fight antibiotic-resistant bacteria, and as for now, phage therapy is essentially performed in combination with antibiotics. Interestingly, combined treatments including phages and a wide range of antibiotics lead to an increased bacterial killing, a phenomenon called phage-antibiotic synergy (PAS), suggesting that antibiotic-induced changes in bacterial physiology alter the dynamics of phage propagation. Using single-phage and single-cell techniques, each step of the lytic cycle of phage HK620 was studied in *E*. *coli* cultures treated with either ceftazidime, cephalexin or ciprofloxacin, three filamentation-inducing antibiotics. In the presence of sublethal doses of antibiotics, multiple stress tolerance and DNA repair pathways are triggered following activation of the SOS response. One of the most notable effects is the inhibition of bacterial division. As a result, a significant fraction of cells forms filaments that stop dividing but have higher rates of mutagenesis. Antibiotic-induced filaments become easy targets for phages due to their enlarged surface areas, as demonstrated by fluorescence microscopy and flow cytometry techniques. Adsorption, infection and lysis occur more often in filamentous cells compared to regular-sized bacteria. In addition, the reduction in bacterial numbers caused by impaired cell division may account for the faster elimination of bacteria during PAS. We developed a mathematical model to capture the interaction between sublethal doses of antibiotics and exposition to phages. This model shows that the induction of filamentation by sublethal doses of antibiotics can amplify the replication of phages and therefore yield PAS. We also use this model to study the consequences of PAS on the emergence of antibiotic resistance. A significant percentage of hyper-mutagenic filamentous bacteria are effectively killed by phages due to their increased susceptibility to infection. As a result, the addition of even a very low number of bacteriophages produced a strong reduction of the mutagenesis rate of the entire bacterial population. We confirm this prediction experimentally using reporters for bacterial DNA repair. Our work highlights the multiple benefits associated with the combination of sublethal doses of antibiotics with bacteriophages.

## Author summary

The viruses that infect bacteria, known as bacteriophages, are present in all biotopes and environments and can help us fight bacterial infections. Phage therapy to treat bacterial infections has been used and documented since the 1920s. However, the antibiotic revolution in the treatment of such infections has completely eclipsed phage therapy in Western countries. The use of phages as therapeutic agents is all the more interesting in the current context as antibiotic-resistant bacteria are increasingly widespread and difficult to treat. Modern therapy increasingly uses phages and antibiotics in combination, which sometimes leads to a beneficial synergy. Understanding the mechanisms by which viral epidemics spread among bacteria in the presence of antibiotics provides information about epidemics in general, as well as clues to optimise treatments for bacterial infections.

## Introduction

Antimicrobial resistance (AMR) is one of the major threats in public health, and a recent study estimates that almost 5 million deaths were associated worldwide with AMR in 2019 [1]. Moreover, in the context of the current COVID-19 pandemic, tackling antibiotics resistance remains a high priority for the World Health Organisation (WHO). The medical community is highly concerned by the increased risk of antibiotics misuse as well as by the decreased concern of worldwide governments to face this major public health issue, one of the consequences of the viral pandemic [2,3]. In this context, phage therapy is more than ever regaining scientific and medical interests after several decades of exclusive use of antibiotics. In Europe, since January 2018, phage magistral preparations have been approved and applied in Belgium [4], whereas in others countries, compassionate usage has been approved in combination with different antibiotics [5,6]. Note that a genetically modified bacteriophage has been successfully used recently to treat an immunocompromised cystic fibrosis young patient dying from a *Mycobacterium abscessus* infection [7]. Interestingly, most recent therapeutic usage combine the use of antibiotics together with specific phage preparations that proved to be more effective in preclinical studies [8,9]. The use of phages in the treatment of infections is currently almost exclusively carried out in combination with antibiotics. Thus, a deep understanding of the interactions occurring in the presence of phages and antibiotics is urgently needed.

Phages and antibiotics combinatorial treatments can lead to three situations: they can behave either synergistically, antagonistically, or without any additional effect compared to independent treatments [10]. Moreover, the resulting effect on the host is highly dependent on the antibiotic class and by the host physiology. It’s been acknowledged by different studies that phage bactericidal efficacy can increase massively when phages and antibiotics were used in combination in clinical practice [9,11], a phenomenon called PAS, for Phage-Antibiotic Synergy [12]. Several laboratories worldwide are studying PAS but most studies are restricted to a limited number of combinations, and rarely focused on the mechanistic elucidation of this effect [10,13,14]. Interestingly, ecological and epidemiological considerations are emerging nowadays as keys to elucidate and predict the consequences of such combinatorial treatments [15]. Moreover, combinatorial treatments not only can modify the killing potential of phages and antibiotics, but can also re-sensitize antibiotic tolerant bacterial strains [16–19]. In addition, this combinatorial approach may also foster the decrease of the antibiotic dosage and reduce the selective pressure on bacteria, thus the potential for antibiotic resistance emergence during therapy [17].

Despite the interest of the medical and ecological scientific communities, very few examples in the literature proposed a mechanism for increased cell killing under combined phage and antibiotic treatments [10,14]. The first suggested mechanism is the delayed lysis hypothesis, which links the burst-size (number of virions produced by a single infected host cell) to the latent period, which is the time between phage adsorption and the release of the first virions [14]. In this work, Kim *et al*. suggested that PAS is the consequence of a prolonged time of particle assembly (latent period) before lysis that increased the yield of virions produced per cell, hence producing an increased number of viruses for the next round of infection. Host lysis requires the production, accumulation, and clusterization of a phage protein named holin that forms holes in the cell membrane. Some antibiotics, such as β-lactams or those inducing the SOS response, promote cell filamentation but this increase in cell membrane surface does not correlate with an increased phage holin production [14]. As a consequence, there are limited amounts of holins to form clusters and holes in the host membrane, hence delaying cell lysis. Strikingly, in a different study it has been shown that under PAS conditions, the lysis time could also be decreased, leading to accelerated lysis while giving rise to a global higher production of virions [12]. This observation is strengthened by studies using phage T4, where mutations in an anti-holin encoding gene (LIN) lead to a more rapid cell lysis but also produce higher virions yields at the population scale [20,21].

In this work we investigated the link between increased phage infection and antibiotic driven bacterial filamentation caused by ceftazidime, cephalexin which belong to the ß-lactam class, and ciprofloxacin, that is a fluoroquinolone. They promote cell elongation through different mechanisms. Past studies on PAS were conducted at the population level and our work emphasizes on the need to investigate PAS at the single cell level to elucidate phage susceptibility in the different bacterial subpopulations elicited by the antibiotic treatment. Accordingly, we developed single-cell as well as single-phage approaches to monitor the critical steps of phage infection using several experimental phage models, such as the temperate phage HK620 and its natural host *Escherichia coli* TD2158 PL4 or the virulent phages T4, T5, and T5 infecting *E*. *coli* MG1655. We show that filamentation-inducing antibiotics are promoting increased phage killing in liquid as well as in solid cultures. We developed a mathematical model to study the influence of sublethal doses of antibiotics on phage-bacteria dynamics. This theoretical framework yields predictions on the synergistic effects of antibiotics and phages on bacterial population growth and on the rise of antibiotic resistance. This joint theoretical-experimental approach elucidates the underlying processes leading to PAS and shows that the combination of phages and antibiotics could have an additional value in terms of lowering the influx of antibiotic resistance mutations.

## Results

### Filamentation-inducing antibiotics act synergistically with phage HK620

**Fig 1:**
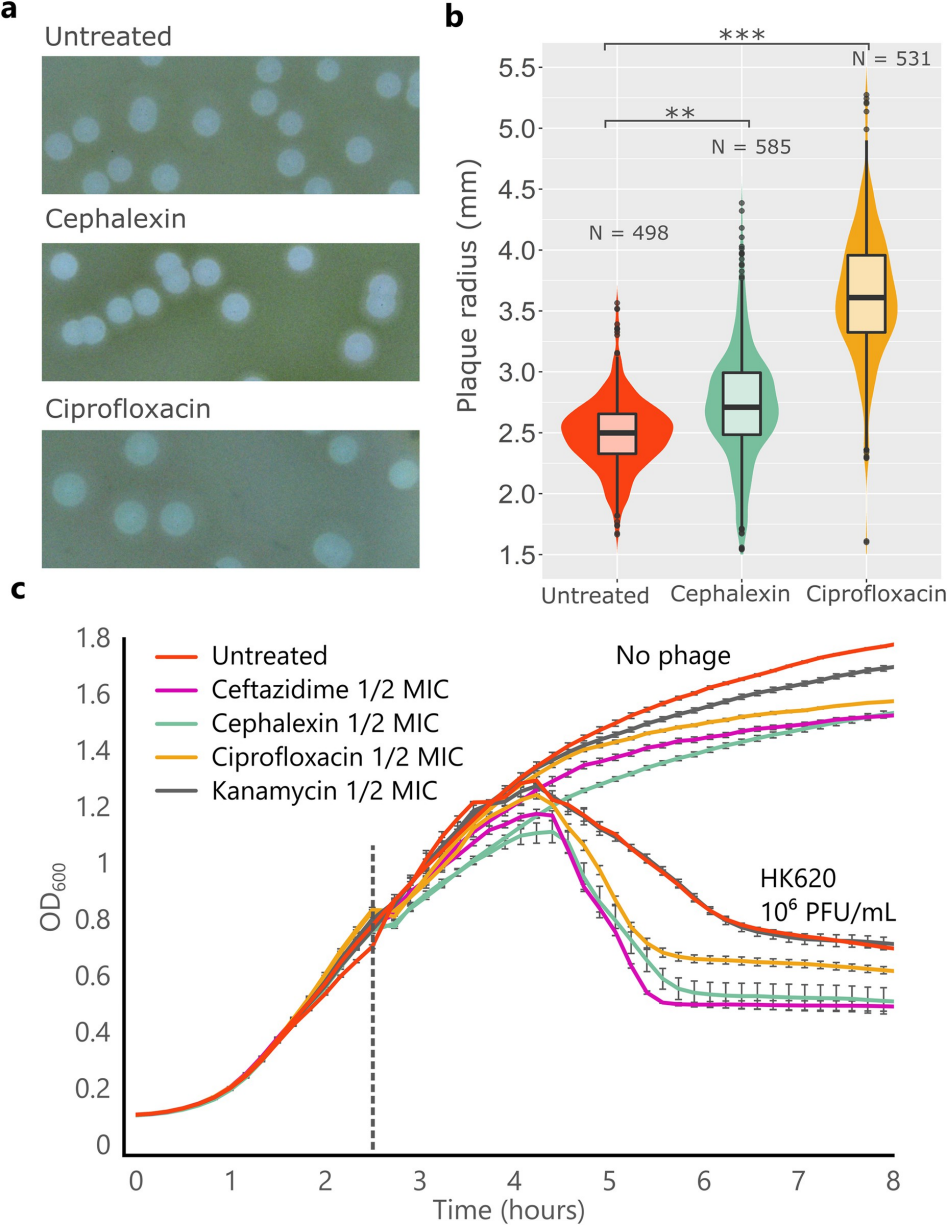
Phage-Antibiotic Synergy (PAS) of phage HK620 and filamentation-inducing antibiotics. **(a)** HK620 lysis plaques from a double agar overlay assay on a lawn of *E*. *coli* TD2158 PL4, with and without filamentation-inducing antibiotics such as cephalexin and ciprofloxacin at ½ MIC. **(b)** Lysis plaque radii comparison between treatments applied in **a**. P values for a two-tailed test of less than 0.001 are denoted with three asterisks, less than 0.01 with two asterisks. **(c)** PAS in liquid culture. The filamentation-inducing antibiotics (ceftazidime, cephalexin and ciprofloxacin), as well as kanamycin, that does not induce filamentation, were present at inoculation time. The vertical dashed line represents the time of phage HK620 addition at a concentration of 10^6^ PFU/mL. Error bars indicate standard error of mean (S.E.M.), N = 4 biological replicates per conditions.

This study addresses the phenomenon of Phage-Antibiotic Synergy, understood as a boost in phage propagation in the presence of antibiotics at low dosages, that otherwise would not lead to significant cell death [12]. PAS measurements were performed in semi-solid and liquid cultures using HK620 natural bacterial host *E*. *coli* TD2158 PL4, which was cured for all resident prophages that proved to be inducible [22,23]. Although HK620 is a temperate phage, it shows a very high spontaneous induction level at 30°C, and behaves fully lytic at 37°C [23]. PAS on a semi-solid culture was tested by measuring lysis plaque radii generated by HK620 on a top agar assay (Fig 1A and 1B). In the presence of either ciprofloxacin or cephalexin used at ½ of the MIC, phage HK620 average plaque radius increased by 32.8% (p < 0.001) and 9.6% (p<0.01), respectively. PAS was also measured on liquid cultures by comparing bacterial optical density reduction (measured at 600 nm, OD_600_) upon phage infection in the presence or absence of antibiotics. We first tested different fractions of the MIC obtained with ciprofloxacin (1/8, ¼, ½) and determined the best concentration to use (S1 Fig). After two hours of incubation with or without antibiotics, the same number of HK620 phages (10^6^ Plaque Forming Units—PFU) was added to each well. All tested concentrations displayed synergy with phage HK620 and this effect was dose-dependent (S1 Fig). We thus chose to use ½ MIC concentration as a standard. In the presence of any of the synergistic antibiotics, including ceftazidime, cephalexin and ciprofloxacin, bacterial killing occurred significantly faster and more efficiently than in the untreated culture as evidenced by the sharpest drop in bacterial population about 90 min after phage addition (Fig 1C). The presence of the antibiotics at sub-lethal concentrations did not impair culture growth, as observed by OD_600_ measurements in the absence of phage. We also included kanamycin that does not induce any morphological change to the cells to the antibiotic panel as a negative control. Indeed, OD_600_ reduction was not observed in the presence of kanamycin (Fig 1C). Together, these results confirm the synergistic behaviour of phage HK620 in combination with filamentation-inducing antibiotics, such as ceftazidime, cephalexin and ciprofloxacin, used at sublethal concentrations (half of the MIC) evidenced an enhanced cell lysis and killing of *E*. *coli* TD2158 PL4 both in liquid and semi-solid media.

### Three filamentation-inducing antibiotics, ceftazidime, cephalexin and ciprofloxacin act synergistically with various phage models

**Fig 2:**
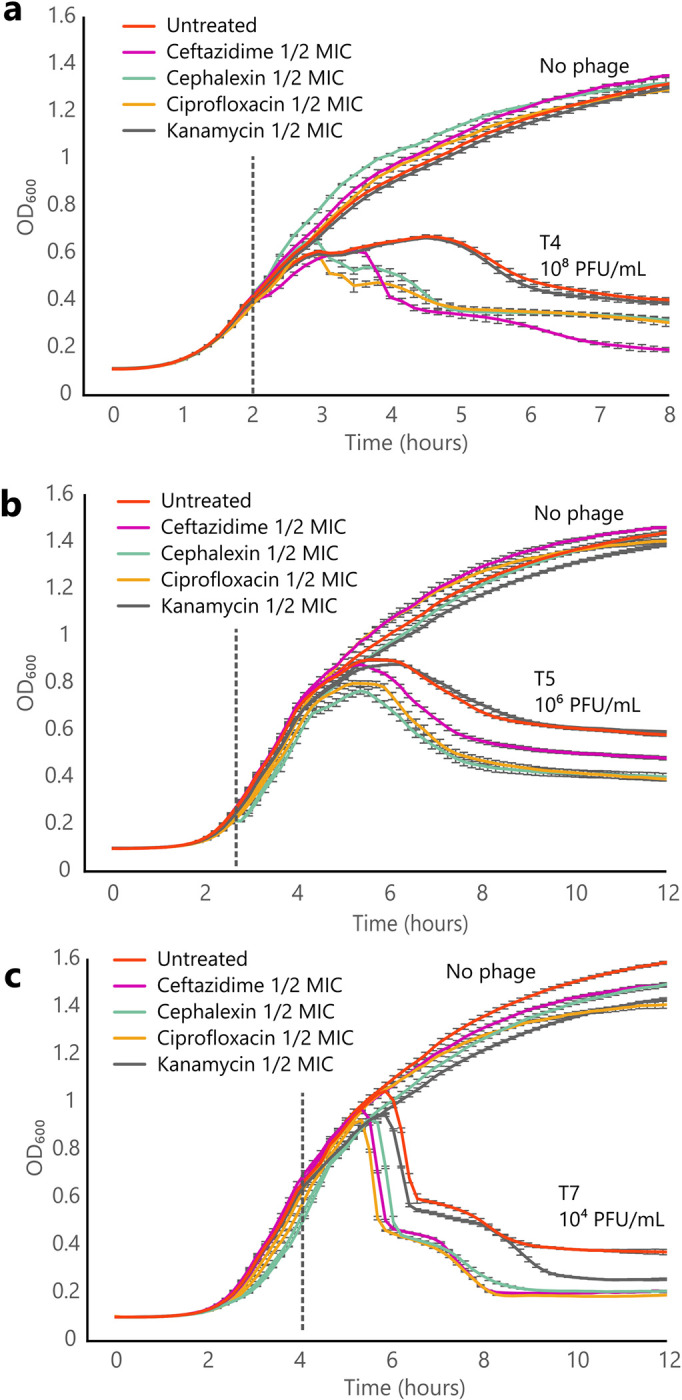
Synergistic profile of phage T4, T5 and T7 on *E*. *coli* MG1655 in the presence of various antibiotics. **(a,b,c)** Lysis of a liquid culture of *E*. *coli* MG1655 by phage T4 **(a)**, T5 **(b)**, and T7 **(c)** at 37°C under agitation 180 rpm. The dotted vertical line represents the time of phage addition. Error bars represent the S.E.M.

In order to generalize the observations we made with phage HK620 and its specific host, we performed synergy assays in liquid cultures with different phages. Fig 2 shows bacteria lysis profiles in the presence of three different virulent phages including T4, T5 and T7, in combination or not with four antibiotics, as well as the control corresponding to the untreated condition. As expected, in the presence of sublethal antibiotic concentrations, OD_600_ measurements show little differences compared to the untreated condition. A synergistic effect was observed in the presence of the three filamentation-inducing antibiotics, namely ceftazidime, cephalexin and ciprofloxacin. As it was the case for phage HK620, synergy was characterized in all cases by an accelerated lysis of the bacterial culture (Fig 2A, 2B and 2C). This acceleration was observed about 30 minutes post-infection for phage T7, and up to two hours for phage T4. In all cases, a reduction of the final values of OD_600_ (post-lysis) was observed. Such synergistic behaviour was not observed in the presence of kanamycin, which does not induce any morphological change on bacterial cells. These results support the role of filamentation in PAS.

### Heterogeneous response to ciprofloxacin and cephalexin creates a subpopulation of filamentous bacteria

One of the landmarks of fluoroquinolone treatment is the induction of bacterial filamentation due to the SOS response activation [24]. β-lactam antibiotics can also cause filamentation, although through a different mechanism, which relies on the disruption of peptidoglycan metabolism via the inhibition of bacterial penicillin binding proteins (PBPs) [25]. In both cases, the number of bacteria that filament and the average cell length were dose-dependent. After testing several concentrations of each antibiotic, we decided to keep half of the MIC as our experimental concentration, since it led to the largest synergistic effect on phage propagation. In the presence of such concentrations of ciprofloxacin or cephalexin, a significant subpopulation of *E*. *coli* TD2158 PL4 fails to divide properly and produce filaments of variable sizes (S2A and S2B Fig). Whereas 99% of the untreated population displayed a cell length below 5.2 μm, cell length increased above 5.2 μm for 30% and 58% of the population in the presence of ciprofloxacin and cephalexin, respectively. The tail of the cell length distribution under these conditions extended up to 12 μm. In both cases, the filaments were composed of a single, continuous cytoplasm harbouring several chromosomes (S2C Fig).

### Single-phage tracking of infection

Since we previously evidenced under PAS conditions we were dealing with heterogenous populations of bacteria, it was therefore important to track infection at the single cell and single phage scale to take account for the heterogeneity of bacterial populations and to detect any tropism of phages for a given subpopulation. To study how phage HK620 interacts with each of the morphologically-different *E*. *coli* subpopulations, we tracked phage adsorption and infection at single-cell level using epifluorescence microscopy. Phage HK620 was transiently labelled with GFP after propagating it in the TD2158 PL4 host producing the plasmid-encoded protein fusion HkbS-GFP. Labelled phages do not encode the fusion on their own genome, allowing to properly observe only a single round of phage adsorption. As HkbS is the major capsid protein (MCP) of phage HK620, we needed to avoid any steric hindrance that could prevent correct virion assembly and stability, we thus introduced a 15-amino acid linker between the MCP and the GFP sequences (see Methods section). For image analysis, we separated elongated from non-elongated subpopulations, the length threshold used to discriminate between regular-size and filamentous cells was set to 5.2 μm since 99% of the untreated *E*. *coli* TD2158 PL4 cells in exponential growth phase fall below this threshold (S2 Fig).

**Fig 3:**
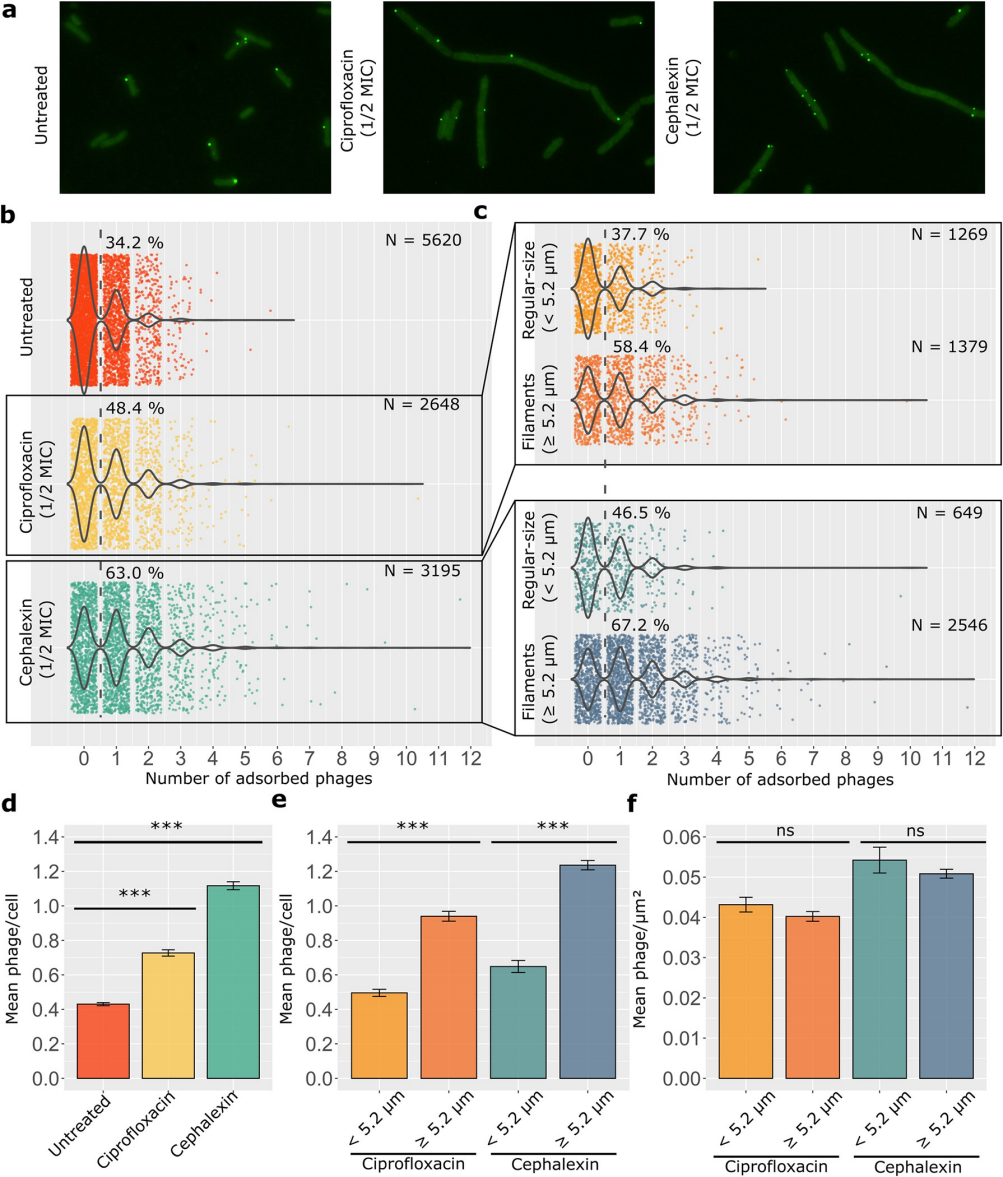
Distribution of HK620 adsorption on *E*. *coli* TD2158 PL4 cells. **(a)** Epifluorescence microscopy of GFP-coated HK620 on the surface of untreated, ciprofloxacin, and cephalexin-treated *E*. *coli* cells. **(b)** Comparison of phage adsorption distribution between untreated, ciprofloxacin, and cephalexin-treated cultures; the dashed lanes and percentages indicate the fraction of cells with one or more phages per cell. **(c)** Comparison of frequencies of adsorption between regular-size and filamentous subpopulations within the ciprofloxacin and cephalexin-treated cultures. Bacterial cells were segmented and fluorescent-phage foci were detected and assigned to the corresponding cell using MicrobeJ, an ImageJ plug-in. Percentages represent the fraction of cells that have adsorbed at least one phage on their surface. **(d)** Mean number of phage adsorption for untreated, ciprofloxacin, and cephalexin-treated cultures. **(e)** Mean number of phages adsorbed to regular-size or filamentous subpopulations within the ciprofloxacin and cephalexin-treated cultures. **(f)** Mean number of phage adsorption per unit of surface for regular-size and filamentous subpopulations within the ciprofloxacin and cephalexin-treated cultures. Error bars indicate standard error of mean (S.E.M.). P values less than 0.001 for a two tailed test are summarised with three asterisks, non-significant differences are marked as “ns”.

Tracking of phage virions by fluorescence microscopy showed that mean HK620 adsorption per cell increased after ciprofloxacin or cephalexin treatment compared to the untreated condition (Fig 3B). As previously theorised [26] it can be observed that phage adsorption follows a Poisson distribution since it takes place at a constant rate and since an individual HK620 adsorption event does not modify the likelihood of further phage adsorption. Under antibiotic treatment, 48.4% of the ciprofloxacin and 63.0% of the cephalexin-treated cells had at least one phage attached to their surface versus 34.2% of the untreated culture (Fig 3B). The presence of at least one phage adsorbed to the cell surface was considered as an indicator of potential infection, since adsorption constitutes the first step of phage replication. To gain a closer look at the effects of antibiotic-induced cell filamentation, we separated each antibiotic-treated population in two subpopulations according to their length (Fig 3C). This allowed us to evaluate the effect of cell length heterogeneity on phage adsorption: For regular-size cells (<5.2 μm), we observed one phage adsorbed per cell for 37.7% (ciprofloxacin) and 46.5% (cephalexin) of this subpopulation. These proportions increased for elongated cells (≥ 5.2 μm) at 58.4% (ciprofloxacin) and 67.2% (cephalexin). Thus, on average, antibiotic-treated cultures display twice (ciprofloxacin) or three times (cephalexin) more phage adsorbed per cell than the untreated cultures (Fig 3D). This difference in phage adsorption can be explained through a rather simple mechanism: since subinhibitory ciprofloxacin and cephalexin concentrations do not impair growth in mass but still inhibits cell division (S3 Fig), a net reduction in cell numbers is observed. Thus, if the number of phages remains constant, an indirect consequence of these antibiotic treatments is an increase of phage/bacteria ratio due to cells that fail to divide. Furthermore, if we focus on the antibiotic-treated cultures, filamentous bacteria adsorb on average twice more phage per cell than their regular-size counterparts (Fig 3E). Together these results show that in a heterogeneous population of bacteria, filaments adsorbed more phages than regular-size cells, whatever the agent causing elongation. Since phage adsorption is proportional to the collision frequency between the phage and its host [27], we hypothesised that the increased level of attachment to filamentous cells was due to their enlarged cell surface. To evaluate this, we calculated the mean HK620 adsorption per unit of surface area between subpopulations (Fig 3F). Our calculations indicated that no significant difference of adsorption per unit of surface between filaments and regular-sized cells occurred upon infection.

**Fig 4:**
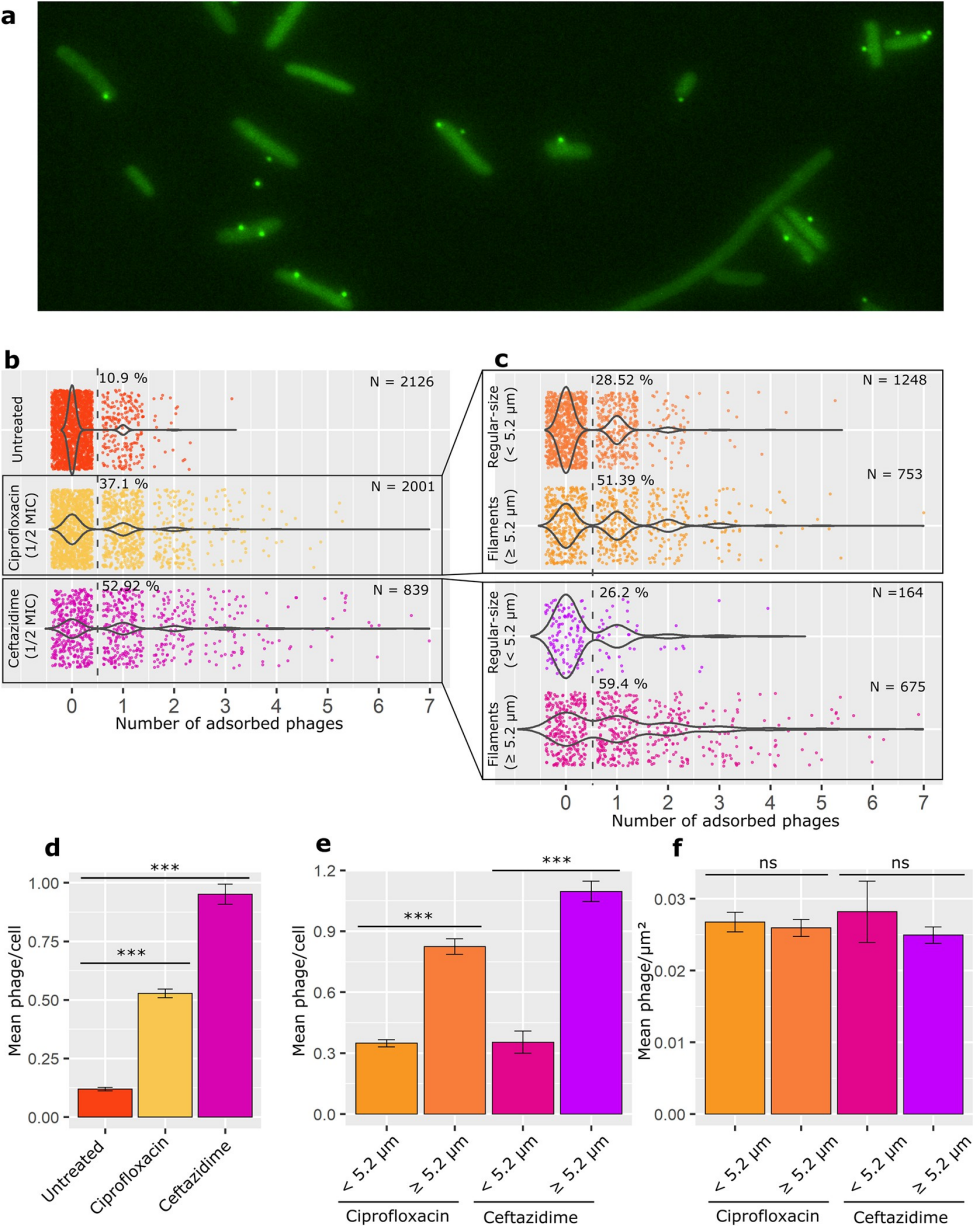
Distribution of phage T5 adsorption on *E*. *coli* MG1655. **(a)** GFP channel of an epifluorescence microscopy image showing the appearance of fluorescent T5 phages (coated with Pb10-GFP fusions) adsorbing to *E*. *coli* MG1655. **(b)** Comparison of phage adsorption distribution between untreated, ciprofloxacin, and ceftazidime-treated cultures; percentages to the right of the dashed lanes indicate the fraction of cells with one or more phages per cell. **(c)** Comparison of frequencies of adsorption between regular-size and filamentous subpopulations within the ciprofloxacin and ceftazidime-treated cultures. Percentages represent the fraction of cells that have adsorbed at least one phage on their surface. **(d)** Mean number of phage adsorption for untreated, ciprofloxacin, and ceftazidime-treated cultures. **(e)** Mean number of phages adsorbed to regular-size or filamentous subpopulations within the ciprofloxacin and ceftazidime-treated cultures. **(f)** Mean number of phage adsorption per unit of surface for regular-size and filamentous subpopulations within the ciprofloxacin and ceftazidime-treated cultures. Error bars indicate standard error of mean (S.E.M.). P values less than 0.001 for a two tailed test are summarised with three asterisks, non-significant differences are marked as “ns”.

In addition, we performed a similar experience using phage T5 coated with a fluorescent version of the head decoration protein Pb10-GFP (Fig 4). Images obtained with T5 show a similar profile as for HK620 (Fig 4A). Moreover, the quantification of the number of phages adsorbed per cell also showed a clear bias of adsorption towards filamenting cells (> 5.2 μm) in the presence of both ciprofloxacin and ceftazidime (Fig 4E). Finally, as for HK620, the mean numbers of phage adsorbed per unit of surface area for regular-size and filamentous subpopulations did not differ in the ciprofloxacin and ceftazidime-treated cultures (Fig 4F).

Conclusively, our results suggest that there is no significant difference of adsorption per unit of surface between filaments and regular-sized cells for both HK620 and T5 phages used in the presence of two different classes of filamentation-inducing antibiotics (Figs 3 and 4). This supports the idea that phage adsorption is proportional to the bacterial size, making it more frequent in longer cells due to an increased probability of phage-host encounter.

### Filaments are infected by HK620 significantly more often than regular-size cells

**Fig 5:**
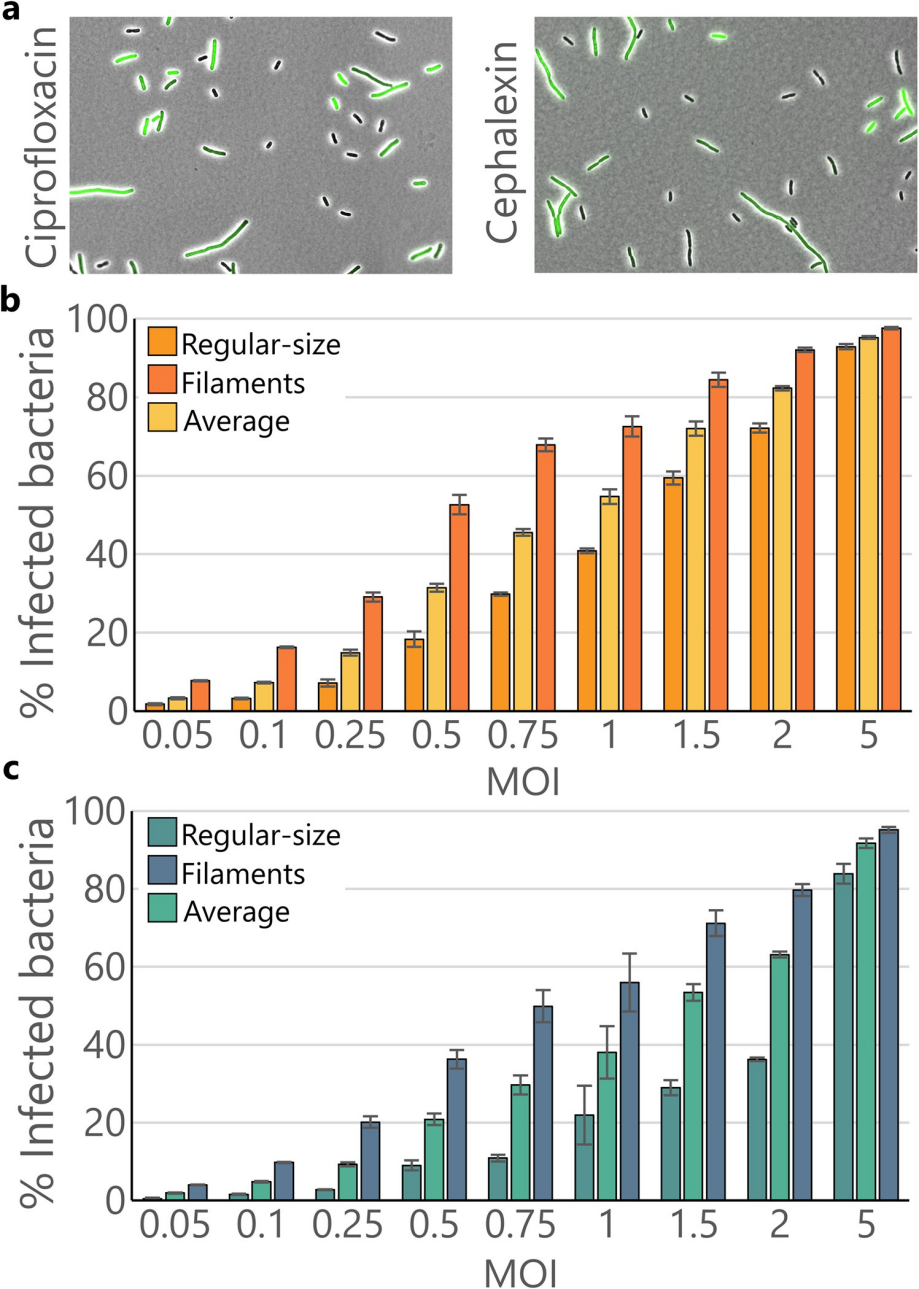
Infection rates of regular-size and filamentous subpopulations within antibiotic-treated cultures. **(a)** Phage-encoded GFP reporter allows to discriminate between naïve and infected cells at time = 20 minutes. Images show HK620 infection on *E*. *coli* TD2158 PL4 at MOI = 1. Antibiotics are present at ½ of the MIC. **(b)** Infection rate of each subpopulation in ciprofloxacin-treated (½ MIC) cultures. **(c)** Infection rate of each subpopulation in cephalexin-treated (½ MIC) cultures. Error bars indicate standard error of mean (S.E.M), N = 3 biological replicates per treatment.

As adsorption is only the first step of a phage productive cycle, we wondered whether the fraction of productive infection was also different between regular-size and filamenting cells. To evaluate this, we used HK620 *hkcEF*::*P*_*rrnB*_*-gfp* [28], a reporter phage that express GFP fluorescence during phage intracellular replication (Fig 5A, see S1 Movie and S4 Fig for the time-lapse analysis of HK620 *hkcEF*::*P*_*rrnB*_*-gfp* infection). The non-compartmentalized GFP fluorescence suggested that filamentous cells share the same cytoplasm and get infected as a single cell. The fraction of infected cells was calculated in the presence and absence of ciprofloxacin or cephalexin and plotted according to the multiplicity of infection (MOI) (Fig 5B and 5C). This MOI was calculated as the average number of phage per cell (PFU/CFU) at the time of infection. The rate of infected cells observed within the filamentous subpopulation was significantly higher than in regular-sized bacteria. These results are in accordance with the observed heterogeneity in phage adsorption. At low initial MOIs such as 0.5 phage/cell, we measured a larger number of fluorescent filaments than regular-sized cells. This confirms the fact that filaments are more prone to interact with phages, due to their enlarged surfaces, than non-filamentous cells. For example, around 50% of elongated cells were infected at a MOI of 0.5 in the presence of ciprofloxacin, whereas it required a MOI of 1–1.5 to reach the same proportion of infected cells in the regular-sized subpopulation. It is only when phages largely outnumber bacteria (initial MOI ≥ 5) that most cells got infected no matter their size.

### Filaments contain more viral factories than regular-size cells

**Fig 6:**
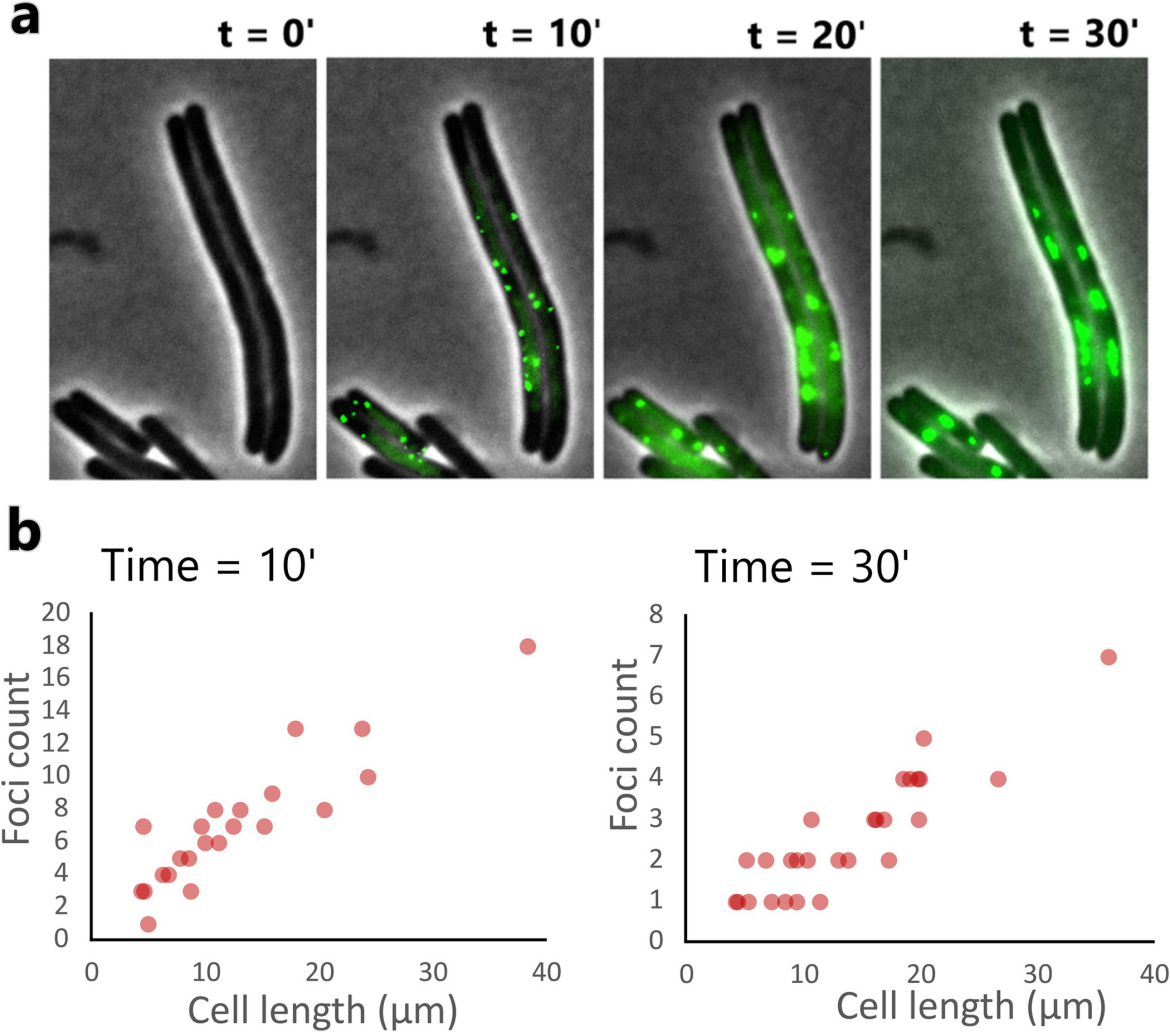
Tracking of phage T7::*parS* DNA in ciprofloxacin-treated *E*. *coli* MG1655. **(a)** Merged phase contrast/epifluorescence microscopy images showing phage T7 DNA replication foci in ciprofloxacin-treated cells over 30 minutes under incubation at 30° C in minimal media. Fluorescence is due to the binding of a host encoded fusion protein ParB-GFP to the sequence *parS* present in T7. **(b)** Quantification of replication foci detected in ciprofloxacin treated samples at 10 min and 30 min post-infection. The detected foci converge in larger viral factories whose number positively correlates with cell length.

In order to provide evidence that phage multiplication massively occurs in elongated cells we decided to label the replicating phage DNA. For this experiment, we used a genetically modified phage T7 where the *parS* sequence has been included in region between *gp11* and *gp12* (see the Materials and Methods section). This engineered phage was then used to infect a *parB-gfp* encoding *E*. *coli* strain. Similarly, to phage HK620 encoding the P*rrnB*::*gfp* that allows a clear visualization of infected cells (Fig 5), the ParB-*parS* system allows to observe and localize viral replication factories within cells (Fig 6). Interestingly, numerous foci were already observed 10 min post-infection, and they tended to merge into bigger foci according to time. We also observed that the number of foci increased as a function of cell length (Fig 6B), thereby showing that multiple viral factories were active in elongated cell cytoplasm’s.

### Cephalexin and ciprofloxacin produce an increase in phage HK620 burst-size

**Fig 7:**
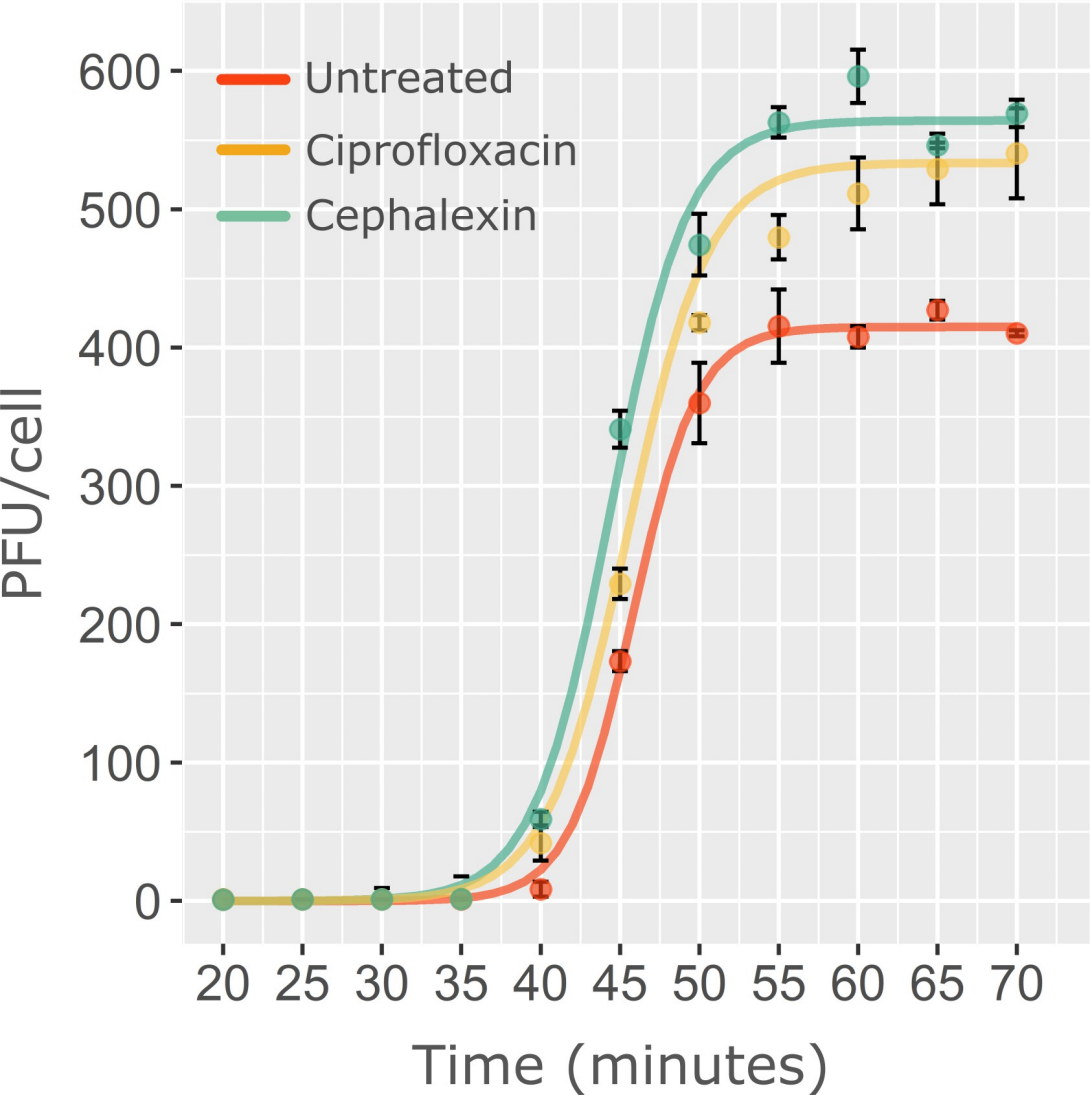
One-Step Growth Curves of phage HK620 on *E*. *coli* TD2158 PL4 at 37°C. HK620 displays a latent period of 40 min and a burst-size of approximately 400 PFU per infected TD2158 PL4 cell. Ciprofloxacin and cephalexin did not significantly modify the latent period, but produced an increased burst size of 28% and 36%, respectively.

An additional question concerns the influence of the antibiotics on the latent period, which is the time frame between the beginning of infection and the release of the phage’s progeny, and the burst-size, which is the number of phages produced per infected cell. We thus measured HK620 burst-size on untreated cells and compared it to ciprofloxacin or cephalexin-treated conditions using the classical One Step Growth Curve (OSGC) experiment (Fig 7). This protocol can only be performed in bulk cultures, making impossible to distinguish differences between the previously observed length subpopulations. This experiment showed that phage HK620 burst size increased by 28% and 36% in the presence of ciprofloxacin or cephalexin, respectively. As we showed that phage infection occurred preferentially in elongated cells (Figs 3–6), phage production should emanate largely from those cells. This increase in productivity is likely beneficial for phage propagation, since more phages are produced per lytic cycle. Surprisingly, the larger burst-sizes were not linked to changes in the latent period. These parameters are usually related since a longer latent period might increase the time available for phage intracellular production. However, our results seem to indicate that a larger burst-size might arise from enhanced rate of phage production in the presence of antibiotics, rather than longer assembly periods.

### Phage HK620 and T7 lyse filaments more often than regular-size cells

**Fig 8:**
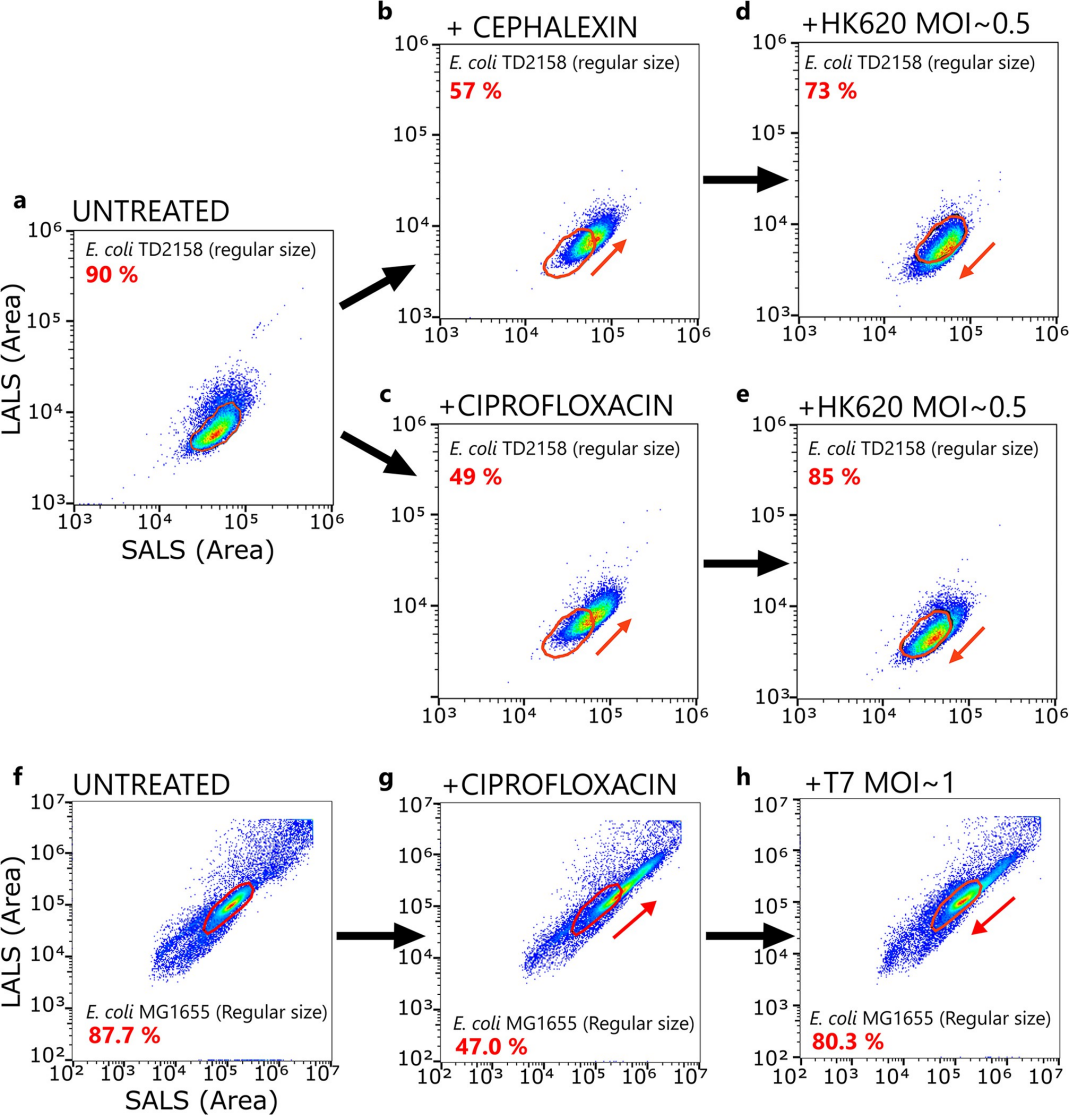
Cytograms of *E*. *coli* populations with or without phage and antibiotic treatment. **(a,b,c,d,e)** *E*. *coli* TD2158 infected by phage HK620 in the presence or absence of cephalexin or ciprofloxacin. **(a)** After 2 h of exponential growth, and before antibiotic treatment. **(b,c)** After 2 h of exponential growth in the presence of cephalexin or ciprofloxacin at half of the MIC concentrations. **(d,e)** After one of round of HK620 lysis at MOI = 0.5. Percentages in red represents the population within the gating (red ellipse). **(f,g,h)** *E*. *coli* MG1655 infected by phage T7 in the presence or absence of ciprofloxacin. **(f)** Cytogram of an *E*. *coli* MG1655 culture after 2 hours of exponential growth. **(g)** Same bacterial strain in the presence of Ciprofloxacin at half the MIC. **(h)** Ciprofloxacin-treated *E*. *coli* MG1655 after 25 min post-infection with phage T7 (approximately one round of lysis), at MOI ~ 1. Percentages in red represents the population within the gating (red ellipse).

**Fig 9:**
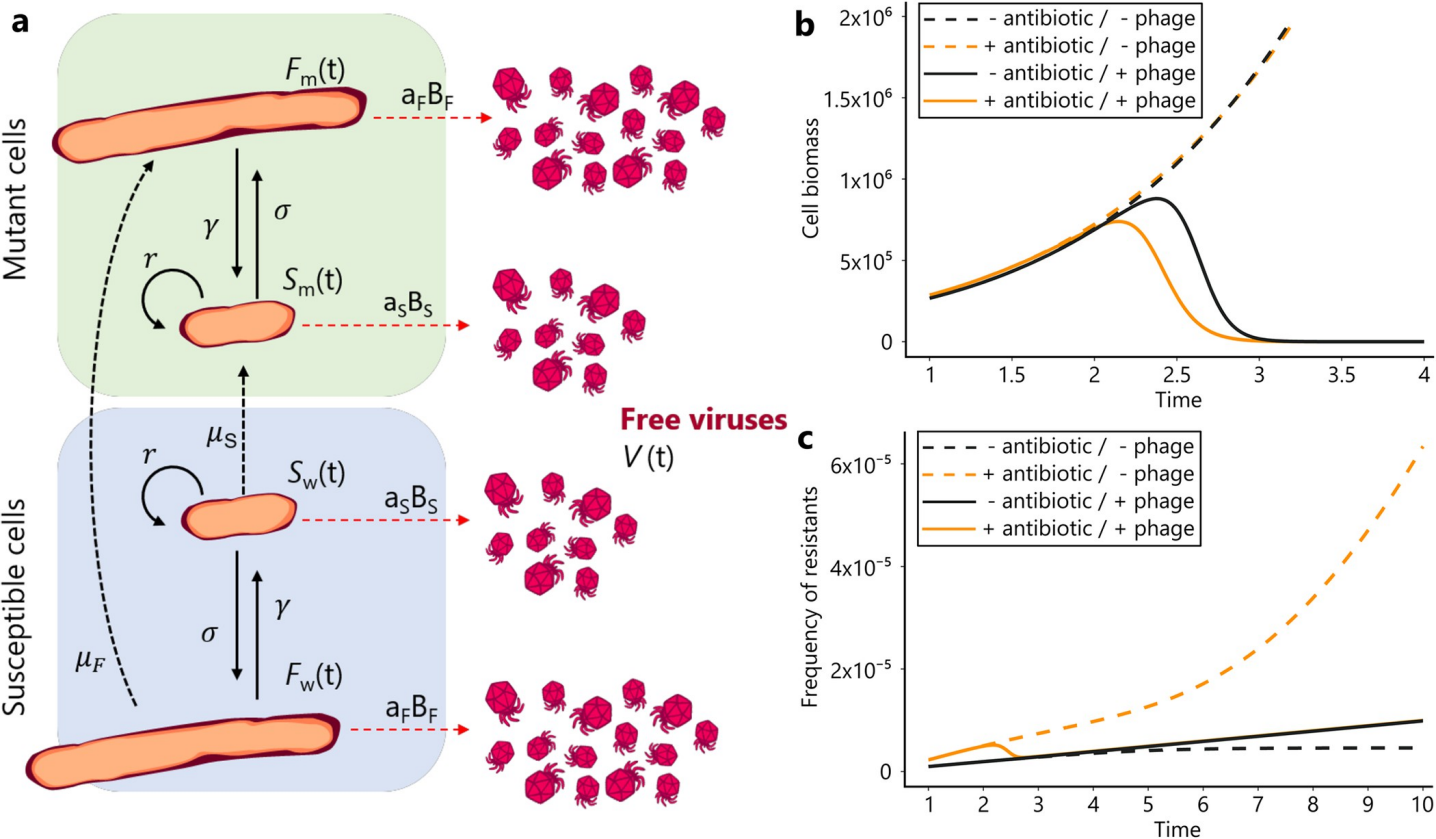
A mathematical model to understand the synergy between antibiotics and phages. **(a)** Schematic representation of the interactions between bacteria and phages in the presence of filamentation-inducing antibiotics (S1 Text, S1 Table). **(b)** Dynamics of the biomass $S+\frac{a_{F}}{a_{S}}F$ of bacteria in the presence of sublethal doses of antibiotics and/or in the presence of phages. **(c)** Dynamics of the frequency of mutant bacteria in the presence of sublethal doses of antibiotics and/or in the presence of phages. The bottom black dashed line indicates the dynamics of the frequency of mutant bacteria in the absence of antibiotics. Note that we obtain the same dynamics if phages are present because phage only affect this dynamic if some filamenting bacteria are present.

In order to spot which subpopulation was effectively lysed upon infection, we performed a flow cytometry analysis after a single round of phage infection. An untreated *E*. *coli* TD2158 culture was analysed and used to create a gating that includes 90% of the population (Fig 8A). As previously stated, this culture consists mostly of regular-size cells (S2 Fig). Upon sublethal antibiotic treatments, a filamentous subpopulation emerged accounting for 43% and 51% of the total population for cephalexin and ciprofloxacin-treated cultures, respectively (Fig 8B and 8C). We then subjected the same antibiotic-treated cultures to a single round of phage lysis before fixation and analysis by flow cytometry. Cytograms showed a neat decrease of the filamentous subpopulations that had appeared upon antibiotic treatments (Fig 8D and 8E). We performed a similar experiment using the fast killer phage T7 and we could observe a similar trend in the lysis profile (Fig 9), demonstrating this effect was not restricted to phage HK620. These results unambiguously show that longer cells are preferentially lysed during infection.

### A mathematical model to understand the synergy between antibiotic and phage treatments

**Fig 10:**
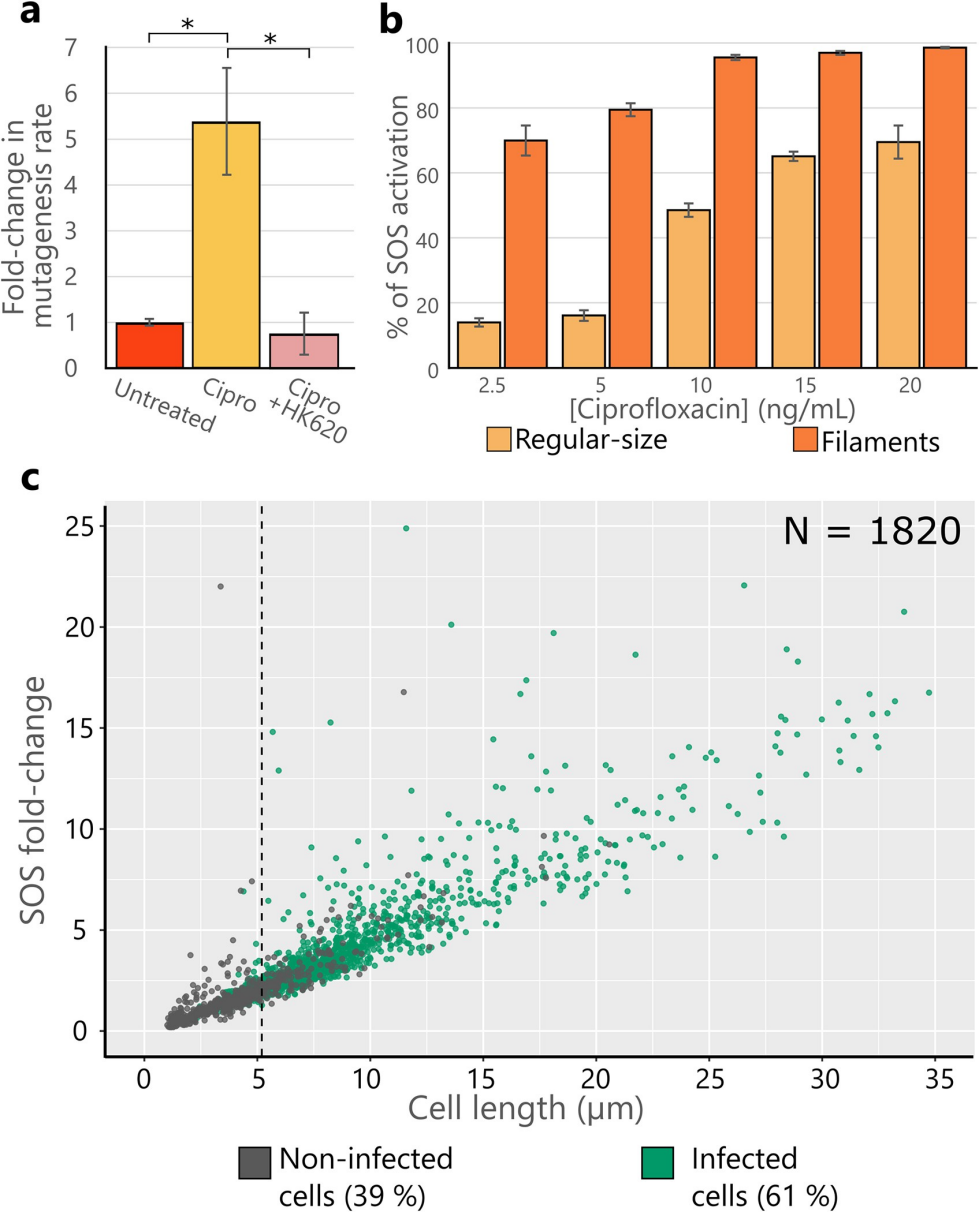
Phage HK620 effect on bacterial mutagenesis and SOS activation in regular-size cells and filaments. **(a)** Fold-increase in mutagenesis rate following ciprofloxacin treatment, and its reduction after the addition of HK620 30 PFU/well at 11 hours post-inoculation. P values of less than 0.05 for a one-tailed test are summarised with a single asterisk. **(b)** The *pcda’*-*gfp* fusion expression was monitored at increasing concentrations of ciprofloxacin in filamentous as well as in non-filamentous subpopulations. The figure shows the percentage of each subpopulation above a threshold defined by the levels of fluorescence found in a ciprofloxacin-free bacterial population. **(c)** Fluorescence fold-change in cells carrying the *pcda’*-*mcherry* reporter versus cell length treated with ciprofloxacin (½ MIC). Percentages in the figure represent the abundance of each subpopulation in the culture. As expected from **(b)**, a strong correlation between cell-length and SOS activation is observed. The dashed vertical line represents the size threshold for filamentation (5.2 μm). Percentages of HK620 *hkcEF*::*PrrnB*-gfp infection on each subpopulation (at MOI = 1) were of 41% and 82% for regular-sized cells and filaments, respectively. The filamentous, hypermutagenic subpopulation was infected more frequently than cells retaining a regular shape that had, contrarily, lower SOS activation rates.

We developed a model to integrate all the effects discussed above on the dynamics of a population of bacteria exposed to a combination of phage and antibiotic treatments (Fig 10 and S1). Bacteria may be exposed to a sublethal dose of antibiotic at rate *σ* which blocks the division of bacteria and induces filamentation of susceptible cells (*S* and *F* refer to the densities of regular size and filamentous cells, respectively). Filamentous bacteria may eventually recover from the filamentous state at rate *γ* and resume to normal cell division. Bacteria may also be exposed to a bacteriophage growing lytically and *V* refers to the density of free viral particles. We show with this model that when we account for the higher adsorption rate on filamentous cells (i.e., when *a_F_*>*a_S_*) we recover the synergistic effect of antibiotic and phage on the total biomass of the bacterial cells (Fig 9B and S1), which accurately describes the experimental results (Figs 2–8).

Our model can also be used to explore the effect of phage predation on the accumulation of mutations in populations exposed to sublethal doses of antibiotics. Indeed, filamentous bacteria are known to launch DNA repair systems and thus to exhibit higher mutation rates [29]. Such an increase in mutation rates increases the likelihood that individual cells acquire mutations that allow them to escape the effects elicited by the antibiotic treatment. Yet, if phage predation is preferentially directed towards filamentous cells (i.e., when *a_F_*>*a_S_*) the frequency of filamentous cells drops (see Figs 8 and S1), which then may reduce the accumulation of mutations in the bacterial population. Our model predicts that even if sublethal doses of antibiotic may trigger mutagenesis, the combination of antibiotic and phage should yield a lower frequency of mutations enabling antibiotic resistance (Fig 9C). We test this prediction in the following section.

### Phage infection decreases SOS-induced mutagenesis

As fluoroquinolones are prone to induce mutagenesis through the SOS-response [30], we wondered if phage addition could modify the mutagenesis rate associated with the ciprofloxacin treatment. For this, we adapted the fluctuation test protocol, which estimates the number of rifampicin resistant mutants (Rif^R^) obtained in the presence of a given stress [31] (see the Methods section for details). First, we verified by OD_600_ measurements, that neither antibiotic nor phage treatment significantly impacted bacterial growth at the concentrations and MOIs employed (S1, S2 and S5A Figs). Although HK620 behaves lytically at 37°C, it remains a temperate phage. Hence, to rule out the occurrence of lysogeny under our experimental conditions we looked at the presence of HK620 integrated in the *E*. *coli* chromosome as previously described [23] at the end of the experiment through colony PCR (S5B Fig). As described recently [32], in the presence of ciprofloxacin lysogeny is highly reduced, since fluoroquinolones are strong inducers of the SOS response, which in many cases can reduce prophage stability. Indeed, 100 CFU obtained from different replicates plated after 8 hours of HK620 infection in the presence of ciprofloxacin were pooled in five groups and screened for HK620 integration. No PCR amplification was observed within any of the pooled colonies (S5C Fig). To evaluate HK620 effect on mutagenesis we measured the number of Rif^R^ colonies that appeared in untreated versus ciprofloxacin-treated cultures after 24 hours. As expected, the mutagenesis rate increased fivefold in the presence of ciprofloxacin, due to error-prone replication occurring upon SOS response activation. We then measured the effect of phage HK620 addition in a ciprofloxacin-treated culture. Strikingly, this rate decreased more than five times when HK620 was added in the presence of the antibiotic (Fig 10A), restoring a mutagenesis rate equivalent to the one in the untreated condition. This result suggests that, in agreement with our theoretical prediction (Fig 9 and S1), while ciprofloxacin increases mutagenesis through the activation of the SOS-response, phage killing seems to reduce this effect. It is remarkable that this effect is achieved even when phages are added in very low doses such as 30 PFU/well (MOI ~10^−7^). To correlate phage infection to SOS-response, we used the *pcda’-gfp* and *pcda’-mcherry* reporter fusions, previously described as an accurate reporter of the SOS-response (Fig 10B and 10C) [33]. As shown above, phage predation occurred preferentially within the elongated subpopulation, which constitutes the majority of the SOS-induced subpopulation. Hence, we propose that phages preferentially target a subpopulation under active DNA repair (prone to mutagenesis). Therefore, the combination of phage and antibiotics may carry two benefits: first, a faster bacterial killing, and second a possible reduction of the influx of antibiotic resistance due to mutagenesis.

## Discussion

### PAS biological significance

Although phage-antibiotic synergy or PAS has been coined by a relatively recent study [12], it has been observed in multiple labs for several decades. In this work, Comeau *et al*. observed using plaque assays that the bactericidal efficacy increases massively when phages and antibiotics are used in combination. However, PAS has been demonstrated to be highly dependent on the appropriate phage and antibiotic combination, therefore empirical determination has become a rule to determine a particular synergy both *in vitro* and *in vivo* [8,18,19,34]. PAS has been observed on both planktonic and biofilm-embedded bacteria, which thus confers a high potential in human health as well as in animal health and agriculture applications. Several laboratories worldwide are studying PAS, but most studies are restricted to a limited number of combinations, and are rarely focusing on the molecular elucidation of this effect [10,13,14]. It is also important to elucidate and predict the consequences of such combinatorial treatments from an epidemiological point of view [15]. Importantly, combinatorial treatments not only increase the killing potential of phages and antibiotics but were also shown to be able to re-sensitize antibiotic resistant bacterial strains in some cases [16–19]. In addition, combined treatments can also induce changes in antibiotic administration habits by decreasing doses, which will reduce selective pressure and thus the potential for the emergence and spread of antibiotic resistance [17].

In a therapeutic context, however, one could question the experimental parameters that most laboratories working on the subject are using. Indeed, PAS has been defined by Comeau *et al*. in a precise zone where antibiotic concentrations are lower than the MIC, because inside the zone of total inhibition, no effect can be observed [12]. Yet, concentrations much higher than the MICs are applied to patient infection sites and it should be considered that MICs determined *in vitro* do not fully correspond to *in vivo* data where bacterial populations as well as the environment are not homogeneous. In the case of ß-lactam antibiotics, for example, the presence of ß-lactamase-producing species could occur and rapidly degrade the antibiotics applied down to sub-inhibitory concentrations [35]. In addition, the *in vivo* pharmacokinetics associated with different antibiotics can lead to a rapid decrease in concentration, which can rapidly fall below the MIC. It should also be noted that antibiotic concentrations fall rapidly after treatment and are therefore compatible with the lower concentrations for which PAS is observed. Another consideration to take into account for *in vivo* treatments, is the effect of antibiotics on resistant strains. It has been shown that resistant strains become filamentous upon antibiotic treatment, even though the concentrations are not reaching the higher MICs that characterize those strains [36].

### Mechanism

The increasing interest of the medical, microbiological and ecological scientific communities is in agreement with the large number of articles studying the effects of combined phage and antibiotic treatments *in vitro* as well as in patients. However, very few examples in the literature proposed a mechanism for increased cell killing under combined treatments [10,14]. The first proposed mechanism is the delayed lysis hypothesis, which links the burst-size (number of virions produced by a single infected host cell) to the latent period, which is the time between phage adsorption and the release of the first virions [14]. In this work, Kim *et al*. suggested that PAS is the consequence of a prolonged time of particle assembly (latent period) before lysis that increased the yield of virions produced per cell. Host lysis requires the production, the accumulation and the clustering of a phage protein named holin that forms holes in the cell membrane. Some antibiotics, such as β-lactams or those inducing the SOS response, promote cell filamentation but this increase in cell membrane surface does not correlate with an increased phage holin production. This leads to limited amounts of holins to localize in clusters and form holes in the host membrane, hence delaying cell lysis. Strikingly, in a different study it has been shown that under PAS conditions, the lysis time could also be decreased, leading to accelerated lysis but also giving rise to a higher production of virions [12]. This observation is strengthened by studies using phage T4, where mutations in an anti-holin encoding gene (LIN) lead to a more rapid cell lysis but also produce higher virions yields [20,21]. However, in the present study, although phage HK620 burst-size significantly increased in the presence antibiotics, the lysis time remained unaffected, even though the antibiotics we used are part of the β-lactam family (cephalexin) or induce the SOS response (ciprofloxacin) (Fig 7). In addition, some studies point to the host membrane fragility in the presence of antibiotics belonging to the β-lactam family that target PBP involved in peptidoglycan synthesis. It was thus proposed that membrane fragility contributed to the decrease of the lysis time [12]. However, this hypothesis would only be valid for β-lactams and does not explain the effect of other classes of antibiotics. The mechanism we describe here applies to two different strains of *E*. *coli* and to a variety of bacteriophages, including HK620, T4, T5, and T7. As synergy with antibiotics inducing filamentation has been described for different organisms [9], this mechanism should be further explored.

We designed a set of experiments that allowed us to dissect phage infection at the single-phage and single-cell levels and identify the subtle changes at various stages of the phage cycle that could not be identified when looking at bulk experiments. We used filamentation-inducing antibiotics at ½ of the MIC, producing populations of heterogeneous size (S2 Fig). Phage infection was then quantified at the phage adsorption step to the bacterial cells (Figs 3 and 4), at the phage genome replication step (Figs 5 and 6) and finally at the lysis step (Fig 8). All these experiments converge to the same conclusion: elongated cells are infected and lysed by phages more often than regular-sized cells due to their enlarged surfaces. This difference in phage-host interaction was previously overlooked using classical bulk methods such as liquid culture or plaque assay that cannot address bacterial population heterogeneity (Figs 1 and S2). Moreover, compartmentalization of phage-encoded *gfp* expression was not observed in infected filaments (Fig 5, S1 Movie), suggesting that cells that fail to divide share the same fate after getting infected by a single phage. Such an effect, which can be thought as killing several cells with one virus, can explain the accelerated eradication of bacteria in liquid cultures. In addition, in our experimental settings and using phage HK620 as a model system, the latency period that reflects the time of lysis did not vary according to the presence of antibiotics (Fig 7). The simplest explanation is that elongated cells catch more phages due to their increased surface (or volume) and that a larger cytoplasm provides more resources to produce and assemble more virions. To date, no technique has been developed yet to determine the burst size of an individual bacterium and future work should aim at developing such technique using high resolution microscopy. Our results show that under these conditions, the adsorption step is critical and that subsequent infection steps follow on from this. This could not have been determined without the single-cell/phage infection techniques we developed.

### Bacterial resistance emergence

A critical aspect of the combined therapy that needs to be considered is the emergence of bacterial resistance to antibiotics or to phages as a consequence of the selective pressure imposed by the treatments. In the recent years it has been shown that phage genomes rarely encode antibiotic resistance genes, yet the transfer of these genes between bacteria by the mean of phages essentially occurs through generalized transduction, i.e. when a phage particle encapsulates random pieces of the host genome that can carry such resistance genes [37]. On the other hand, antibiotic and phage resistance can occur through mutagenesis that may generate altered versions of a variety of bacterial proteins leading to resistance or modify their production. On the phage-resistance side, such mutations generally affect receptor molecules (lipopolysaccharide or membrane proteins) that become non-permissive to recognition or phage genome ejection [38]. On the antibiotic-resistance side, a variety of mutations can lead to antibiotic resistance according to the antibiotic’s molecular targets (efflux pumps, PBPs, transcription and translation machineries). As an example, mutations in *rpoB*, encoding the β-subunit of the RNA-polymerase, lead to rifampicin resistance, which is the principle used by fluctuation tests to estimate mutagenesis rate [39]. Sublethal concentrations of fluoroquinolones as well as β-lactams tend to increase mutagenesis [30,40]. Our results confirmed that fluoroquinolone-induced elongated cells are SOS-activated and more prone to mutagenesis (Fig 10). Since the SOS-activated population is a target of choice for phages, we thus wondered if phages could alter the mutagenesis rate. We performed an adapted fluctuation protocol to quantify mutation rates, and our results suggested that the addition of phages reduced the mutagenesis induced by ciprofloxacin (Fig 10). Hence, a combined treatment is expected to reduce *in vivo* resistance acquisition and produce a more efficient therapy than antibiotics or phages applied separately. However, even though several clues point toward this direction, this dual effect remains hypothetical, and we need to continue our experimental work to fully demonstrate it. In addition, it has been shown in a number of recent studies that acquired phage resistance could lead to antibiotics resensitization [38,41–43], reinforcing the need to investigate resistance dynamics to both phage and antibiotics under combined treatments.

### Model predictions and experimental validation

As pointed above, the existence of PAS depends on a specific combination of antibiotic and phage. In the present work we focused on antibiotics of two different classes (fluoroquinolone and β-lactam) that both produce cell filamentation (S2 Fig). We contend that the induction of filamentation by sublethal doses of antibiotics may drive PAS. To explore this hypothesis, we developed a mathematical model where the exposition to antibiotic yields filamenting cells (Fig 9). A phage spreading in a bacteria population exposed to sublethal doses of antibiotics will thus have to exploit different types of cell subpopulations. The above experimental results identify the influence of these different cells on the successive steps of the phage life cycle. Our model shows that the higher adsorption rate on filamenting cells affects the composition of the cell population and amplifies the reduction in the population biomass because larger cells are preferentially killed by the phage. Besides, this mechanistic model also captures the influence of phage predation on the reduction in the influx of mutant cells.

**Fig 11:**
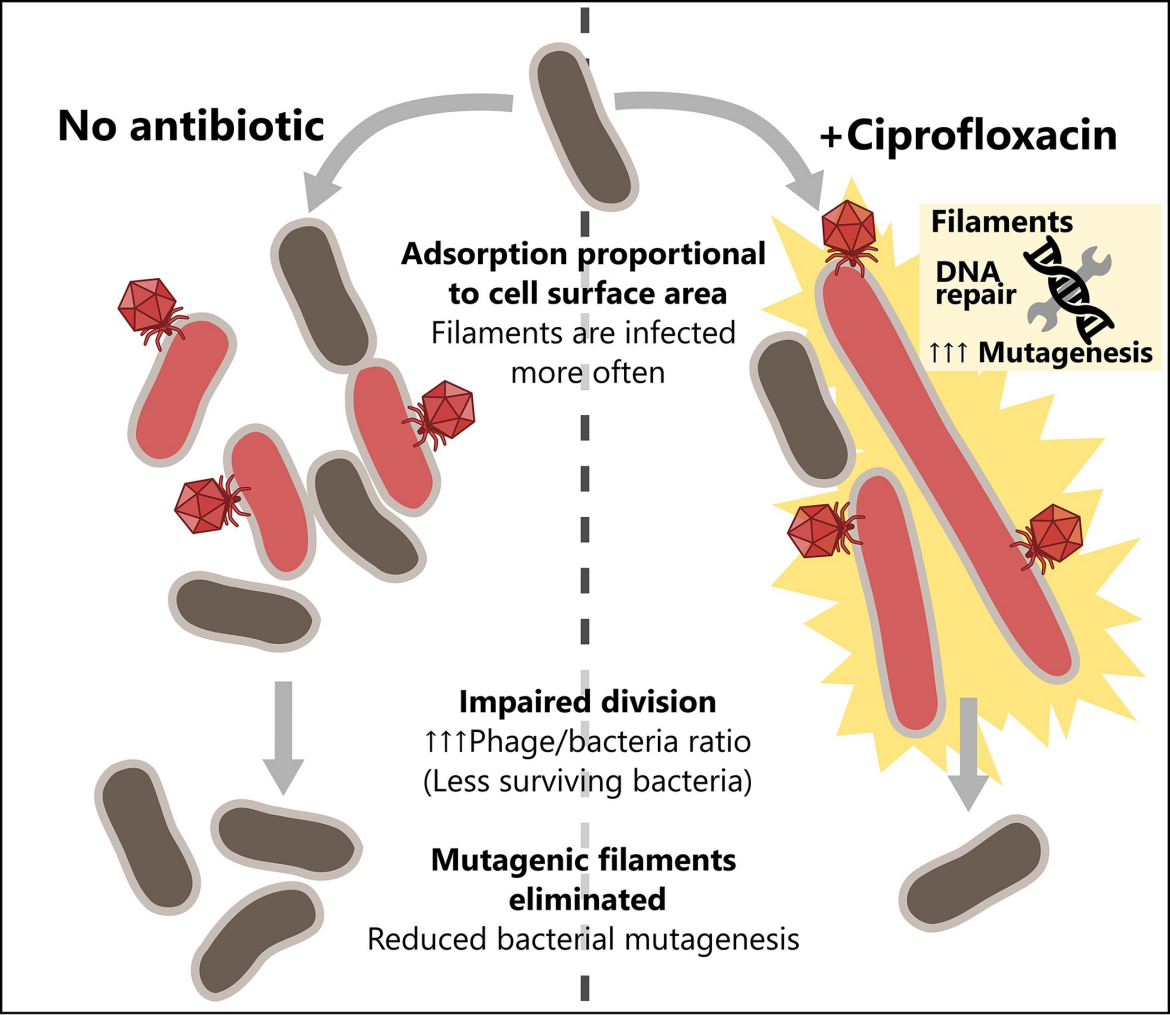
A graphical model for ciprofloxacin-enhanced killing by phage HK620, which prevents bacterial mutagenesis.

Hence, our mechanistic description on the effects of antibiotics on the morphology and the physiology of bacterial cells and their impact on phage dynamics provides a deeper understanding of the effects of combining phage and antibiotic treatments. Combination therapy may provide two distinct beneficial effects (Fig 11). First, a purely demographic effect where combination reduces the biomass of bacterial population (the classical PAS effect). Second, an evolutionary effect where combination reduces the influx of mutations in the bacteria population. Both effects could help maximize the efficacy of phage therapy. Yet, an evaluation of the efficacy of combination therapy needs to account for other processes such as the evolution of resistance to phage and to the antibiotic used in combination. In addition, our model could be readily extended to take into consideration the effects of the immune response of the host on the dynamics of the bacteria [15].

## Materials and methods

### Bacterial strains, phages and culture conditions

Phage HK620 and its host *E*. *coli* TD2158 were originally provided by J. Clark and S. Barbirz [22,44,45]. Strain TD2158 PL4 was obtained after curation of inducible prophages using mitomycin C [23]. The engineered phage HK620 *hkcEF*::*P*_*rrnB*_*-gfp* was constructed in a previous work [28]. The bacterial host *E*. *coli* MG1655 used in this study originated from P. Genevaux’s collection. Bacteriophage T4 belongs to a laboratory collection. Bacteriophage T5 was provided by P. Boulanger team. Bacteriophage T7 was provided by the DSMZ collection. Unless otherwise indicated, bacterial cultures were carried out in Lysogeny Broth (LB) with 1.5% agar for solid media. Phage propagation was performed by infection of a log-phase bacterial culture at 37°C, under 180 rpm agitation until culture clearing. Phages were collected by filtration of the lysate supernatant and further purified by centrifugation [46].

### Minimum inhibitory concentration determination

To determine which antibiotic concentrations impaired significantly *E*. *coli* TD2158 PL4 growth, OD_600_ curves were performed at 37°C under 180 rpm agitation for 16 hours using a 96-well microplate reader (Spark multimode microplate reader, Tecan). Bacteria were inoculated at OD_600_ = 0.025 in LB medium with ½ serial dilutions of antibiotics. MIC was designed as the lowest concentration with at least 15% of reduction in the area under the growth curve. The maximum antibiotic concentration that allowed undisturbed growth was considered as ½ of the MIC.

### Phage HK620 latent-period determination

In order to set up every further experiment, HK620 latent-period was determined through the One-Step Growth Curve method (Fig 7). A log-phase *E*. *coli* TD2158 PL4 culture was infected at MOI < 0.001. After 15 min incubation, 100 μL samples were taken and plated at intervals of 5 minutes until 70 minutes post-infection, and lysis plaques counted.

### Phage-Antibiotic Synergy measurements

PAS measurements in liquid cultures were carried out by OD_600_ measurements in a 96-well microplate. Briefly, a log-phase *E*. *coli* TD2158 PL4 culture was diluted to a final OD_600_ = 0.025. Subinhibitory antibiotic concentrations were added at this time. Replicates of 200 μL of each condition were dispensed in each well. After two hours of incubation at 37°C under 180 rpm agitation, the same number of phages (phage titer PFU/mL) was added to each replicate and lysis was observed by the reduction in OD_600_. Conversely, PAS measurements in semi-solid cultures were performed using the double agar overlay assay. For this, 10 mL of a log-phase culture of *E*. *coli* TD2158 PL4 at OD_600_ = 1 were infected with a final titer of 10^3^ PFU/mL of phage HK620 and incubated at 37°C under 180 rpm agitation. Twenty-five minutes post-infection, 100 μL of this culture was mixed with 3 mL of soft-LBA (0.75% agar) and plated over a 20 mL bottom layer of 1.5% LBA on a petri dish. Antibiotics were added at sublethal concentrations (half of the MIC) in the bottom layer. Plates containing 100 PFU each were imaged using a digital camera (Nikon D5300). Lysis plaque diameter was measured using a homemade software designed by Leon Espinosa (available upon request) and average plaque diameter was plotted for each condition.

### Antibiotic effect on division time and cell surface

A log-phase *E*. *coli* TD2158 PL4 culture (OD_600_ = 1) was diluted by 1:1000. Two μL of this dilution were added to an Ibidi μ-Dish 35 mm high microscopy dish (IBIDI, Martinsried, Germany) and squeezed with 1 mm thick 1% agarose-LB pad. Antibiotics were added to the pad according to the previously determined sublethal concentrations. Bacteria were imaged on an inverted phase-contrast microscope (model) thermo-controlled at 37° C. For each condition, three to five individual cells were selected at time zero and time-lapse imaged with 5 minutes intervals for 3 h. Accurate bacterial segmentation was carried out using the Misic tool [47]. For each condition, cell number and surface fold-change were plotted over time.

### GFP-coated HK620 and T5

To avoid stability problems related to a genomic fusion of GFP to HK620 major capsid protein HkbS, a transient GFP labelling was carried out by propagating the phage on an *E*. *coli* TD2158 PL4 strain carrying the fusion encoded by plasmid pUC18-P*rplU*-*hkbS*-*gfp*. Similarly, T5 labelling was carried out by propagating the phage in an *E*. *coli* MG1655 strain carrying the decoration capsid protein Pb10 fused to GFP (plasmid pUC18-P*rplU*-*pb10-gfp*). These strains provide GFP-labelled capsid proteins that incorporates randomly into the capsid during phage assembly together with the unlabelled phage-encoded HkbS for HK620 or Pb10 for T5. As the fusions are not phage-encoded, they allow monitoring of adsorption without additional GFP synthesis once the genome is injected into cells lacking the aforementioned plasmids. To obtain GFP-labelled phages cells were grown to OD_600_ = 1 and infected with 10^6^ PFU/mL of phages at 37° C, 180 rpm. After complete lysis (3–4 hours) cell debris were pelleted by centrifugation at 4000 rpm and the supernatant filtered through a 0.2 μm syringe filter to keep only the labelled phage fraction. Homogenous fluorescent labelling of HK620 and T5 was obtained using this protocol.

### Single-cell infection

An overnight culture of *E*. *coli* TD2158 PL4 was diluted to final OD600 = 0.025 in 10 mL of LB medium, and supplemented with the appropriate sublethal antibiotic concentrations when required. At OD_600_ ≈ 0.8, phages were added to reach a final MOI between 0.1 to 10. Thirty minutes post-infection aliquots were sampled and fixed by diluting 1:1 in PBS buffer containing 4% paraformaldehyde to stop phage replication and prevent cell lysis. For adsorption analysis, unabsorbed phages were washed by centrifugation followed by resuspension of the pellet in PBS- 2% PFA. For all samples, the mix was pipetted onto a coverslip, gently squeezed under a 1 mm thick 1% agarose pad, and directly imaged on an inverted epifluorescence microscope (Nikon TiE) using an oil immersion 100X NA 1.45 objective. Images were acquired using a cooled camera (Hamamatsu Orca Fusion). Acquisition was carried out using Nikon’s NIS-Element software.

### Quantification of T7::*parS* replication foci

A phage T7 carrying a copy of the *parS* sequence between the genes gp11 and gp12 was obtained by recombining the wild type phage with a high copy number plasmid pUC18. This plasmid carried the recombination substrate that included the *parS* sequence and the marker gene *trxA* flanked by homology regions. Briefly an *E*. *coli* MG1655 strain carrying this construction was lysed by the wild type T7 and the recombinant progeny was selected and purified using an *E*. *coli* MG1655 *ΔtrxA*, the bacterial TrxA protein being essential for T7 viral cycle [48].

To study T7 DNA replication, an *E*. *coli* MG1655 strain carrying the *parB*-*gfp* fusion on plasmid pFH2973 under a Plac promoter was used [49]. An exponential culture of this strain growing at 30°C in the presence of ciprofloxacin (1/2 MIC), and induced with 100 μM IPTG was infected with T7::*parS* at MOI~5. The culture was immediately placed on a coverslip, gently squeezed under a 1 mm thick 1% agarose pad, and directly imaged on an inverted epifluorescence microscope (Nikon TiE) using an oil immersion 100X NA 1.45 objective. Time-lapse recording was carried out under incubation at 30°C, imaging every minute. Images were acquired using a cooled camera (Hamamatsu Orca Fusion). Acquisition was carried out using Nikon’s NIS-Element software.

### SOS response induction reporter

To compare the level of SOS activation between regular-size cells and filaments, *E*. *coli* TD2158 PL4 cells harbouring the SOS activation reporter plasmid p*cda’*-*gfp* (Norman et al., 2005) were grown in the presence of increasing ciprofloxacin concentrations, ranging from 2.5 to 20 ng/mL. Two hours post-inoculation (mid-log phase) cells were fixed in 1:1 PBS buffer 4% PFA and observed under the microscope. Morphological parameters and GFP fluorescence intensity were measured for each individual bacterium. To evaluate the frequency of HK620 *hkcEF*::*P*_*rrnB*_*-gfp* infection in SOS-triggered cells the *gfp* reporter gene in p*cda’*-*gfp* was replaced by *mcherry* using the SphI/HindII sites flanking the *gfp* gene. Two hours post-inoculation in the presence of ciprofloxacin (½ MIC), *E*. *coli* TD2158 PL4 /*pcda’-mcherry* cells were infected with HK620 *hkcEF*::*P*_*rrnB*_*-gfp* at a MOI of 1. Infection was interrupted after 30 minutes by diluting the samples 1:1 in PBS buffer PFA 4%. The SOS reporter (p*cda’*-*mcherry*) and HK620 *hkcEF*::*P*_*rrnB*_*-gfp* infection were imaged using the red and green fluorescence channels, respectively.

### Image analysis

Cell image analysis was performed using MicrobeJ [50]. Cell shape parameters were directly measured from phase-contrast microscopy. For phage adsorption quantification, automated foci detection was carried out using the maxima foci function of MicrobeJ, after background subtraction and thresholding the GFP channel to isolate individual fluorescent phages. To quantify phage infection of HK620 *hkcEF*::*P*_*rrnB*_*-gfp* the integrated fluorescence of each cell was measured in the GFP channel. Statistical analysis was conducted in R [51] and figures were produced using the package ggplot2 [52]. To calculate average phage adsorption per unit of surface, *E*. *coli* shape was considered as a cylinder with two half spheres on each extremity. Bacterial length (*L*), measured from pole to pole, and average width (*d*) was determined for each bacterium using the image analysis software MicrobeJ. We then used these measurements to approximate bacterial cell surface through the following equation: π*d*(L-d) + 4*π*(d/2)^*2*^. The first term represents the surface of the cylinder of length (*L-d*), and the second, the surface of the two hemispheres of radius (*d*/2) at each pole.

### Susceptibility to phage lysis through flow-cytometry

A log-phase culture of *E*. *coli* TD2158 PL4 carrying plasmid pP_*rplU*_-*gfp* was infected with HK620 at MOIs ranging from 0.1 to 1. Lysis was allowed to take place only once and the infection was stopped 60 min post-infection by fixing the culture with an equal volume of 4% PFA solution in PBS 1X. A similar protocol was carried out for phage T7 using *E*. *coli* MG1655/ pP_rplU_-*gfp* as host and stopping the infection 20 minutes after the beginning of the infection. Samples were analysed using the compact flow cytometer A50-micro (Apogee Flow Systems, UK) equipped with an argon ion laser (Asbly, wavelength excitation 488 nm, 50 mV) and specific fluorescence filter set (Green (Gn): 535/35 nm, Orange (Or): 585/20 nm and Red (Rd): >610 nm). Calibration beads (Apogee Flow Systems, 1 μm, excitation 488 nm and a broad fluorescence emission in the Gn, Or and Rd channels, 5,000 event/μl^-1^) were used as a standard. Each sample was run in triplicate. Data were acquired in Log scale using PC control v3.40 and histogram v110.0 softwares (Apogee Flow Systems) and analysed with FlowJoV10 software (TreeStarInc).

### Mutation rates measurements

The frequency of Rif^R^ CFU after 20 hours of growth was determined as follows. A log-phase OD_600_ = 1 culture was diluted 1:10^6^ (final concentration 200–500 CFU/mL) and 100 μL were dispensed in each well of a U-shaped bottom 96-well microplate (Nunclon Delta-Treated, U-Shaped-Bottom Microplate, Nunc). Antibiotics, if used, were added at ½ of the MIC at this point. The plate was covered with a sealing tape and incubated at 37°C, 180 rpm, in a humidity cassette to minimise culture evaporation. After 20 h of incubation, the total volume (100 μL) of 84 wells was plated onto separate LBA plates supplemented with 75 μg/mL rifampicin. The remaining 12 wells were serially diluted and plated on non-selective plates to count the total number of CFU. Mutation rates were estimated by the MSS-MLE algorithm provided by the FALCOR calculator (https://lianglab.brocku.ca/FALCOR/). For the experiments in which we assessed phage effect in Rif^R^ mutant frequencies, HK620 phages were added in all wells at 11 h post inoculation with a final titer of 4x10^3^ PFU/well. Each condition was repeated at least three times.

### Dryad DOI

https://doi.org/doi:10.5061/dryad.3ffbg79q8 [53]

## Supporting information

S1 Movie Fluorescence increase upon HK620 *hkcEF*::P*rrnB*-*gfp* infection. Time-lapse recording of *E*. *coli* TD2158 PL4 cells infected with phage HK620 *hkcEF*::P*rrnB*-*gfp* at MOI = 1. Recording of the infection started at time = 10 min. after phage addition and proceeded for 45 minutes with capture intervals of 5 minutes.
(MP4)

S1 Fig Accelerated bacterial killing in the presence of increasing ciprofloxacin concentrations. **(a)** Phase-contrast microscopy images of E. coli TD2158 PL4 cultures grown at increasing ciprofloxacin concentration after 2 hours of treatment at 37° C. The percentage of filaments (cells larger than 5.2 μm) in each sample was of 1.6%, 4.8%, 15.3% and 23.9% for the untreated, 1/8, 1/4, and 1/2 MIC of ciprofloxacin, respectively. **(b)** Lysis curves of phage HK620 in the presence of increasing ciprofloxacin concentrations. Dotted vertical line represents the time of phage addition. N = for each condition, six independent curves were performed.
(TIF)

S2 Fig Effect of subinhibitory antibiotic concentrations on *E*. *coli* TD2158 PL4 morphology. **(a)** Phase-contrast microscopy images of exponential growing *E*. *coli* after 2 hours post-inoculation in the presence of filamentation-inducing antibiotics. **(b)** Cell-length distribution of *E*. *coli* population under different treatments. Mean cell length was of 2.85 μm, 5.16 μm, and 7.59 μm for the untreated, ciprofloxacin-treated and cephalexin-treated samples, respectively. Percentages represent the filamentous subpopulation, here considered with a length equal or higher than 5.2 μm. P values of less than 0.001 for a two tailed test are summarised with three asterisks. **(c)** DAPI staining of each treatment showing the distribution of the bacterial nucleoids within the cytoplasm.
(TIF)

S3 Fig Tracking of *E*. *coli* TD2158 PL4 growth and division rates through phase-contrast microscopy. **(a)** Average number of fully-segmented cells in an *E*. *coli* microcolony over time at 37°C, starting from a single cell. Average generation times were of 17:50 min, 20:25 min and 21:30 min for the untreated, ciprofloxacin and cephalexin conditions respectively. **(b)** Average surface fold-change in the same microcolonies. **(c)** Binary mask of the fully segmented microcolonies obtained through MiSiC.
(TIF)

S4 Fig GFP-fluorescence build-up in HK620 *hkcEF*::*P*_*rrn*_*B-gfp* infected cells. Comparison of fluorescence intensity fold-change over time between infected and uninfected cells in S1 Movie. *E*. *coli* TD2158 PL4 and phage HK620 *hkcEF*::*P*_*rrn*_*B-gfp* were mixed at MOI = 1 at time = 0 minutes. Intensity was measured for N > 15 bacteria belonging to each group. Vertical-dashed lines represent the lysis of the fluorescent cells.
(TIF)

S5 Fig HK620 behaves fully lytic in the presence of ciprofloxacin. **(a)** TD2158 PL4 growth curves untreated, in the presence of ciprofloxacin (½ MIC), or with both ciprofloxacin (½ MIC) and phage HK620 (30 PFU/well). The dashed line represents the time of phage addition (time = 11 hours). **(b)** Schematic representation of primer design to screen for HK620 integration. If the prophage is present, a fragment of 264 bp will be amplified. **(c)** The resulting colony-PCR on pooled clones recovered after 9 hours of infection (time = 20 hours) revealing the absence of the integrated phage (lanes 1 to 5) and a positive control of a TD2158 PL4 HK620 lysogen (lane 6). M = molecular weight marker.
(TIF)

S1 Text The supporting text includes a detailed description of the mathematical model to understand the synergy between antibiotics that promote filamentation and phages summarized in Fig 9. (DOCX)

S1 Table List of parameters used in the mathematical model of PAS and their definition. The values indicated in the tables were used to obtain the curves in Fig 9B and 9C.
(XLSX)

## References

[1] Murray CJ, Ikuta KS, Sharara F, Swetschinski L, Aguilar GR, Gray A, et al. Global burden of bacterial antimicrobial resistance in 2019: a systematic analysis. The Lancet. 2022;0. doi: 10.1016/S0140-6736(21)02724-0 35065702 PMC8841637

[2] Lucien MAB, Canarie MF, Kilgore PE, Jean-Denis G, Fénélon N, Pierre M, et al. Antibiotics and antimicrobial resistance in the COVID-19 era: Perspective from resource-limited settings. Int J Infect Dis. 2021;104: 250–254. doi: 10.1016/j.ijid.2020.12.087 33434666 PMC7796801

[3] Ruiz J. Enhanced antibiotic resistance as a collateral COVID-19 pandemic effect? J Hosp Infect. 2021;107: 114–115. doi: 10.1016/j.jhin.2020.11.010 33217492 PMC7670892

[4] Djebara S, Maussen C, De Vos D, Merabishvili M, Damanet B, Pang KW, et al. Processing Phage Therapy Requests in a Brussels Military Hospital: Lessons Identified. Viruses. 2019;11: 265. doi: 10.3390/v11030265 30884879 PMC6466067

[5] Abedon ST, Danis-Wlodarczyk KM, Alves DR. Phage Therapy in the 21st Century: Is There Modern, Clinical Evidence of Phage-Mediated Efficacy? Pharm Basel Switz. 2021;14: 1157. doi: 10.3390/ph14111157 34832939 PMC8625828

[6] Ferry T, Kolenda C, Briot T, Souche A, Lustig S, Josse J, et al. Past and Future of Phage Therapy and Phage-Derived Proteins in Patients with Bone and Joint Infection. Viruses. 2021;13: 2414. doi: 10.3390/v13122414 34960683 PMC8708067

[7] Dedrick RM, Guerrero-Bustamante CA, Garlena RA, Russell DA, Ford K, Harris K, et al. Engineered bacteriophages for treatment of a patient with a disseminated drug-resistant Mycobacterium abscessus. Nat Med. 2019;25: 730–733. doi: 10.1038/s41591-019-0437-z 31068712 PMC6557439

[8] Gordillo Altamirano FL, Kostoulias X, Subedi D, Korneev D, Peleg AY, Barr JJ. Phage-antibiotic combination is a superior treatment against Acinetobacter baumannii in a preclinical study. eBioMedicine. 2022;80: 104045. doi: 10.1016/j.ebiom.2022.104045 35537278 PMC9097682

[9] Łusiak-Szelachowska M, Międzybrodzki R, Drulis-Kawa Z, Cater K, Knežević P, Winogradow C, et al. Bacteriophages and antibiotic interactions in clinical practice: what we have learned so far. J Biomed Sci. 2022;29: 23. doi: 10.1186/s12929-022-00806-1 35354477 PMC8969238

[10] Gu Liu C, Green SI, Min L, Clark JR, Salazar KC, Terwilliger AL, et al. Phage-Antibiotic Synergy Is Driven by a Unique Combination of Antibacterial Mechanism of Action and Stoichiometry. mBio. 2020;11. doi: 10.1128/mBio.01462-20 32753497 PMC7407087

[11] Diallo K, Dublanchet A. Benefits of Combined Phage–Antibiotic Therapy for the Control of Antibiotic-Resistant Bacteria: A Literature Review. Antibiotics. 2022;11: 839. doi: 10.3390/antibiotics11070839 35884092 PMC9311689

[12] Comeau AM, Tétart F, Trojet SN, Prère M-F, Krisch HM. Phage-Antibiotic Synergy (PAS): beta-lactam and quinolone antibiotics stimulate virulent phage growth. PloS One. 2007;2: e799. doi: 10.1371/journal.pone.0000799 17726529 PMC1949050

[13] Chaudhry WN, Concepción-Acevedo J, Park T, Andleeb S, Bull JJ, Levin BR. Synergy and Order Effects of Antibiotics and Phages in Killing Pseudomonas aeruginosa Biofilms. PLoS ONE. 2017;12. doi: 10.1371/journal.pone.0168615 28076361 PMC5226664

[14] Kim M, Jo Y, Hwang YJ, Hong HW, Hong SS, Park K, et al. Phage-Antibiotic Synergy via Delayed Lysis. Appl Environ Microbiol. 2018;84. doi: 10.1128/AEM.02085-18 30217844 PMC6210123

[15] Rodriguez-Gonzalez RA, Leung CY, Chan BK, Turner PE, Weitz JS. Quantitative Models of Phage-Antibiotic Combination Therapy. mSystems. 2020;5. doi: 10.1128/mSystems.00756-19 32019835 PMC7002117

[16] Cohan FM, Zandi M, Turner PE. Broadscale phage therapy is unlikely to select for widespread evolution of bacterial resistance to virus infection. Virus Evol. 2020;6: veaa060. doi: 10.1093/ve/veaa060 33365149 PMC7744382

[17] Morrisette T, Kebriaei R, Lev KL, Morales S, Rybak MJ. Bacteriophage Therapeutics: A Primer for Clinicians on Phage-Antibiotic Combinations. Pharmacotherapy. 2020;40: 153–168. doi: 10.1002/phar.2358 31872889

[18] Segall AM, Roach DR, Strathdee SA. Stronger together? Perspectives on phage-antibiotic synergy in clinical applications of phage therapy. Curr Opin Microbiol. 2019;51: 46–50. doi: 10.1016/j.mib.2019.03.005 31226502

[19] Tagliaferri TL, Jansen M, Horz H-P. Fighting Pathogenic Bacteria on Two Fronts: Phages and Antibiotics as Combined Strategy. Front Cell Infect Microbiol. 2019;9: 22. doi: 10.3389/fcimb.2019.00022 30834237 PMC6387922

[20] Chen Y, Young R. The Last *r* Locus Unveiled: T4 RIII Is a Cytoplasmic Antiholin. Christie PJ, editor. J Bacteriol. 2016;198: 2448–2457. doi: 10.1128/JB.00294-16 27381920 PMC4999926

[21] Paddison P, Abedon ST, Dressman HK, Gailbreath K, Tracy J, Mosser E, et al. The roles of the bacteriophage T4 r genes in lysis inhibition and fine-structure genetics: a new perspective. Genetics. 1998;148: 1539–1550. doi: 10.1093/genetics/148.4.1539 9560373 PMC1460109

[22] Dhillon TS, Poon AP, Chan D, Clark AJ. General transducing phages like Salmonella phage P22 isolated using a smooth strain of Escherichia coli as host. FEMS MicrobiolLett. 1998;161: 129–133. doi: 10.1111/j.1574-6968.1998.tb12938.x 9561740

[23] Menouni R, Champ S, Espinosa L, Boudvillain M, Ansaldi M. Transcription termination controls prophage maintenance in Escherichia coli genomes. Proc Natl Acad Sci U S A. 2013;110: 14414–14419. doi: 10.1073/pnas.1303400110 23940369 PMC3761637

[24] Ysern P, Clerch B, Castańo M, Gibert I, Barbé J, Llagostera M. Induction of SOS genes in *Escherichia coli* and mutagenesis in *Salmonella typhimurium* by fluoroquinolones. Mutagenesis. 1990;5: 63–66. doi: 10.1093/mutage/5.1.63 2158613

[25] Kocaoglu O, Carlson EE. Profiling of β-Lactam Selectivity for Penicillin-Binding Proteins in Escherichia coli Strain DC2. Antimicrob Agents Chemother. 2015;59: 2785–2790. doi: 10.1128/AAC.04552-14 25733506 PMC4394777

[26] Stopar D. Modeling bacteriophage population growth. In: Abedon ST, editor. Bacteriophage Ecology: Population Growth, Evolution, and Impact of Bacterial Viruses. Cambridge: Cambridge University Press; 2008. pp. 389–414. doi: 10.1017/CBO9780511541483.018

[27] Storms ZJ, Sauvageau D. Modeling tailed bacteriophage adsorption: Insight into mechanisms. Virology. 2015;485: 355–362. doi: 10.1016/j.virol.2015.08.007 26331682

[28] Vinay M, Franche N, Grégori G, Fantino J-R, Pouillot F, Ansaldi M. Phage-Based Fluorescent Biosensor Prototypes to Specifically Detect Enteric Bacteria Such as E. coli and Salmonella enterica Typhimurium. PLoS ONE. 2015;10: e0131466. doi: 10.1371/journal.pone.0131466 26186207 PMC4506075

[29] Pribis JP, García-Villada L, Zhai Y, Lewin-Epstein O, Wang AZ, Liu J, et al. Gamblers: An Antibiotic-Induced Evolvable Cell Subpopulation Differentiated by Reactive-Oxygen-Induced General Stress Response. Mol Cell. 2019;74: 785–800.e7. doi: 10.1016/j.molcel.2019.02.037 30948267 PMC6553487

[30] Bos J, Zhang Q, Vyawahare S, Rogers E, Rosenberg SM, Austin RH. Emergence of antibiotic resistance from multinucleated bacterial filaments. Proc Natl Acad Sci. 2015;112: 178–183. doi: 10.1073/pnas.1420702111 25492931 PMC4291622

[31] Luria SE, Delbrück M. MUTATIONS OF BACTERIA FROM VIRUS SENSITIVITY TO VIRUS RESISTANCE. Genetics. 1943;28: 491–511. doi: 10.1093/genetics/28.6.491 17247100 PMC1209226

[32] Al-Anany AM, Fatima R, Hynes AP. Temperate phage-antibiotic synergy eradicates bacteria through depletion of lysogens. Cell Rep. 2021;35: 109172. doi: 10.1016/j.celrep.2021.109172 34038739

[33] Norman A, Hestbjerg Hansen L, Sørensen SJ. Construction of a ColD cda Promoter-Based SOS-Green Fluorescent Protein Whole-Cell Biosensor with Higher Sensitivity toward Genotoxic Compounds than Constructs Based on recA, umuDC, or sulA Promoters. Appl Environ Microbiol. 2005;71: 2338–2346. doi: 10.1128/AEM.71.5.2338-2346.2005 15870320 PMC1087587

[34] Akturk E, Oliveira H, Santos SB, Costa S, Kuyumcu S, Melo LDR, et al. Synergistic Action of Phage and Antibiotics: Parameters to Enhance the Killing Efficacy Against Mono and Dual-Species Biofilms. Antibiotics. 2019;8: 103. doi: 10.3390/antibiotics8030103 31349628 PMC6783858

[35] Sheu C-C, Lin S-Y, Chang Y-T, Lee C-Y, Chen Y-H, Hsueh P-R. Management of infections caused by extended-spectrum β–lactamase-producing Enterobacteriaceae: current evidence and future prospects. Expert Rev Anti Infect Ther. 2018;16: 205–218. doi: 10.1080/14787210.2018.1436966 29402125

[36] Kjeldsen TS, Sommer MO, Olsen JE. Extended spectrum β-lactamase-producing Escherichia coli forms filaments as an initial response to cefotaxime treatment. BMC Microbiol. 2015;15: 63. doi: 10.1186/s12866-015-0399-3 25888392 PMC4367913

[37] Enault F, Briet A, Bouteille L, Roux S, Sullivan MB, Petit M-A. Phages rarely encode antibiotic resistance genes: a cautionary tale for virome analyses. ISME J. 2017;11: 237–247. doi: 10.1038/ismej.2016.90 27326545 PMC5315482

[38] Mangalea MR, Duerkop BA. Fitness Trade-Offs Resulting from Bacteriophage Resistance Potentiate Synergistic Antibacterial Strategies. Infect Immun. 2020;88: e00926–19. doi: 10.1128/IAI.00926-19 32094257 PMC7309606

[39] Garibyan L, Huang T, Kim M, Wolff E, Nguyen A, Nguyen T, et al. Use of the rpoB gene to determine the specificity of base substitution mutations on the Escherichia coli chromosome. DNA Repair. 2003;2: 593–608. doi: 10.1016/s1568-7864(03)00024-7 12713816

[40] Gutierrez A, Laureti L, Crussard S, Abida H, Rodríguez-Rojas A, Blázquez J, et al. β-lactam antibiotics promote bacterial mutagenesis via an RpoS-mediated reduction in replication fidelity. Nat Commun. 2013;4: 1610. doi: 10.1038/ncomms2607 23511474 PMC3615471

[41] Canfield GS, Chatterjee A, Mangalea MR, Sheriff EK, Keidan M, McBride SW, et al. Lytic bacteriophages facilitate antibiotic sensitization of Enterococcus faecium. bioRxiv. 2020; 2020.09.22.309401. doi: 10.1101/2020.09.22.309401 PMC8092871 33649110

[42] Engeman E, Freyberger HR, Corey BW, Ward AM, He Y, Nikolich MP, et al. Synergistic Killing and Re-Sensitization of Pseudomonas aeruginosa to Antibiotics by Phage-Antibiotic Combination Treatment. Pharm Basel Switz. 2021;14: 184. doi: 10.3390/ph14030184 33668899 PMC7996583

[43] Kortright KE, Chan BK, Koff JL, Turner PE. Phage Therapy: A Renewed Approach to Combat Antibiotic-Resistant Bacteria. Cell Host Microbe. 2019;25: 219–232. doi: 10.1016/j.chom.2019.01.014 30763536

[44] Broeker NK, Gohlke U, Müller JJ, Uetrecht C, Heinemann U, Seckler R, et al. Single amino acid exchange in bacteriophage HK620 tailspike protein results in thousand-fold increase of its oligosaccharide affinity. Glycobiology. 2013;23: 59–68. doi: 10.1093/glycob/cws126 22923442

[45] Clark AJ, Inwood W, Cloutier T, Dhillon TS. Nucleotide sequence of coliphage HK620 and the evolution of lambdoid phages. JMolBiol. 2001;311: 657–679. doi: 10.1006/jmbi.2001.4868 11518522

[46] Clavijo-Coppens F, Ginet N, Cesbron S, Briand M, Jacques M-A, Ansaldi M. Novel Virulent Bacteriophages Infecting Mediterranean Isolates of the Plant Pest Xylella fastidiosa and Xanthomonas albilineans. Viruses. 2021;13: 725. doi: 10.3390/v13050725 33919362 PMC8143317

[47] Panigrahi S, Murat D, Le Gall A, Martineau E, Goldlust K, Fiche J-B, et al. Misic, a general deep learning-based method for the high-throughput cell segmentation of complex bacterial communities. Xiao J, Storz G, Hensel Z, editors. eLife. 2021;10: e65151. doi: 10.7554/eLife.65151 34498586 PMC8478410

[48] Genomewide screens for Escherichia coli genes affecting growth of T7 bacteriophage—PubMed. [cited 15 Jun 2023]. Available: https://pubmed-ncbi-nlm-nih-gov.insb.bib.cnrs.fr/17135349/10.1073/pnas.0609428103 PMC1748173 17135349

[49] Nielsen HJ, Li Y, Youngren B, Hansen FG, Austin S. Progressive segregation of the Escherichia coli chromosome. Mol Microbiol. 2006;61: 383–393. doi: 10.1111/j.1365-2958.2006.05245.x 16771843

[50] Ducret A, Quardokus EM, Brun YV. MicrobeJ, a tool for high throughput bacterial cell detection and quantitative analysis. Nat Microbiol. 2016;1: 16077. doi: 10.1038/nmicrobiol.2016.77 27572972 PMC5010025

[51] R Core Team. R: A language and environment for statistical computing. Vienna, Austria: R Foundation for Statistical Computing; 2021. Available: https://www.R-project.org/

[52] Wickham H. ggplot2: Elegant Graphics for Data Analysis. Springer-Verlag New York. 2016.

[53] Bulssico J, Papukashvili I, Espinosa L, Gandon S, Ansaldi M. Phage-antibiotic synergy: Cell filamentation is a key driver of successful phage predation. 2023. https://doi.org/doi:10.5061/dryad.3ffbg79q8 10.1371/journal.ppat.1011602 PMC10519598 37703280

