# Supplementary figures and images for: Phage-antibiotic synergy: Cell filamentation is a key driver of successful phage predation
Source: PLoS Pathog. 2023 Sep 13;19(9):e1011602. doi: 10.1371/journal.ppat.1011602 (PMC10519598; doi:10.1371/journal.ppat.1011602)

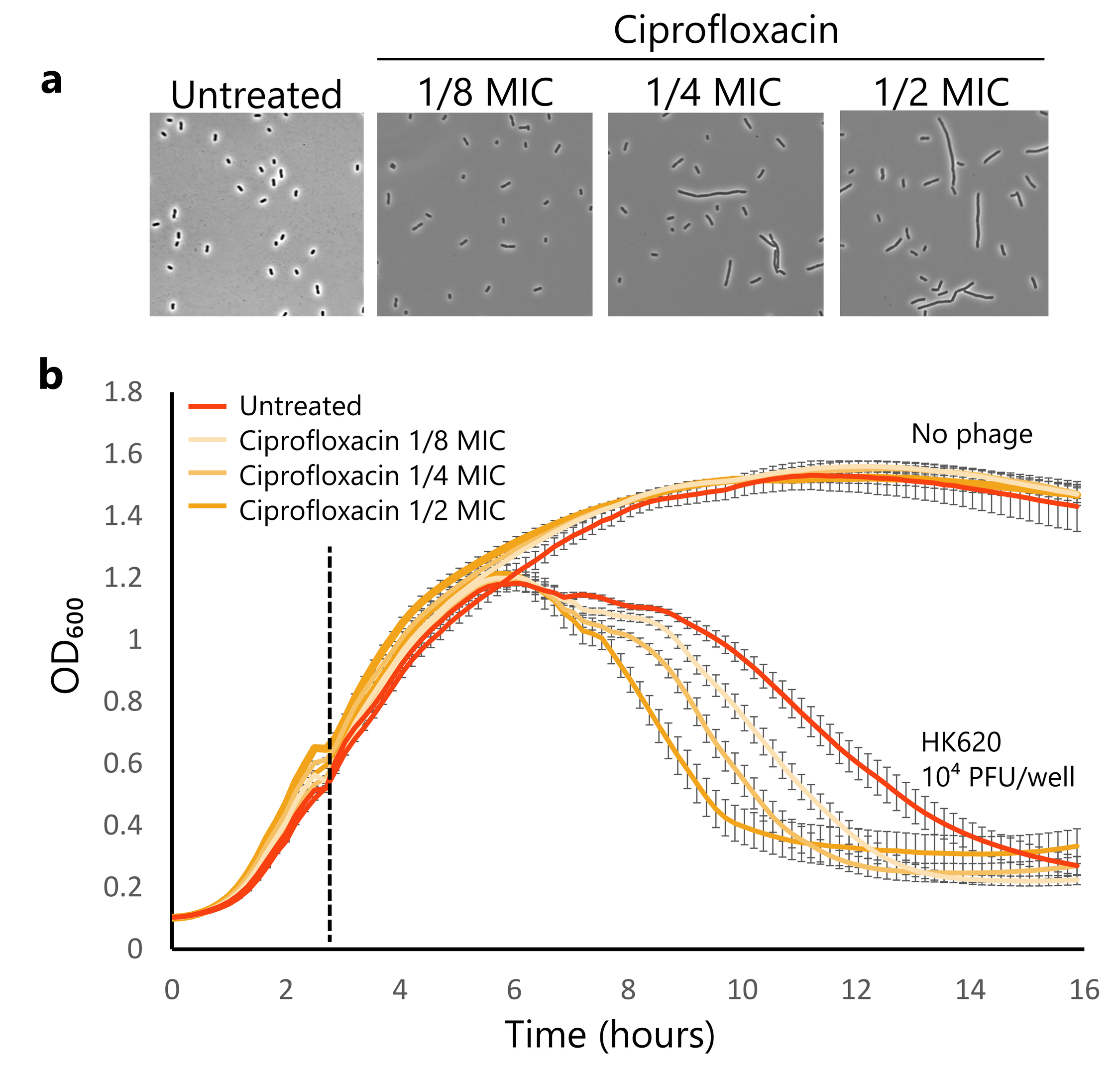

Supplement: S1 Fig — (a) Phase-contrast microscopy images of E. coli TD2158 PL4 cultures grown at increasing ciprofloxacin concentration after 2 hours of treatment at 37° C. The percentage of filaments (cells larger than 5.2 μm) in each sample was of 1.6%, 4.8%, 15.3% and 23.9% for the untreated, 1/8, 1/4, and 1/2 MIC of ciprofloxacin, respectively. (b) Lysis curves of phage HK620 in the presence of increasing ciprofloxacin concentrations. Dotted vertical line represents the time of phage addition. N = for each condition, six independent curves were performed. (TIF) [file ppat.1011602.s002.tif]

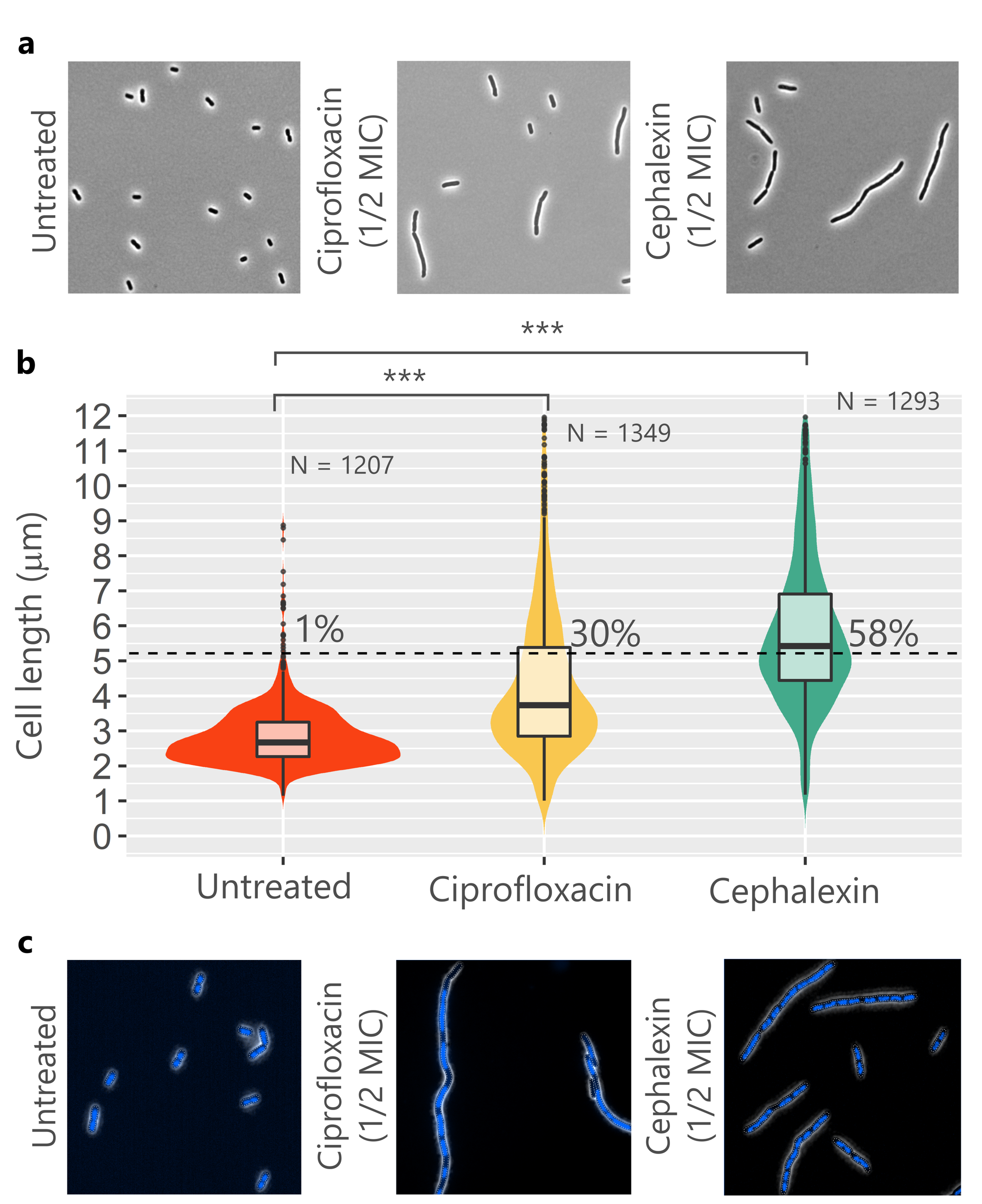

Supplement: S2 Fig — (a) Phase-contrast microscopy images of exponential growing E. coli after 2 hours post-inoculation in the presence of filamentation-inducing antibiotics. (b) Cell-length distribution of E. coli population under different treatments. Mean cell length was of 2.85 μm, 5.16 μm, and 7.59 μm for the untreated, ciprofloxacin-treated and cephalexin-treated samples, respectively. Percentages represent the filamentous subpopulation, here considered with a length equal or higher than 5.2 μm. P values of less than 0.001 for a two tailed test are summarised with three asterisks. (c) DAPI staining of each treatment showing the distribution of the bacterial nucleoids within the cytoplasm. (TIF) [file ppat.1011602.s003.tif]

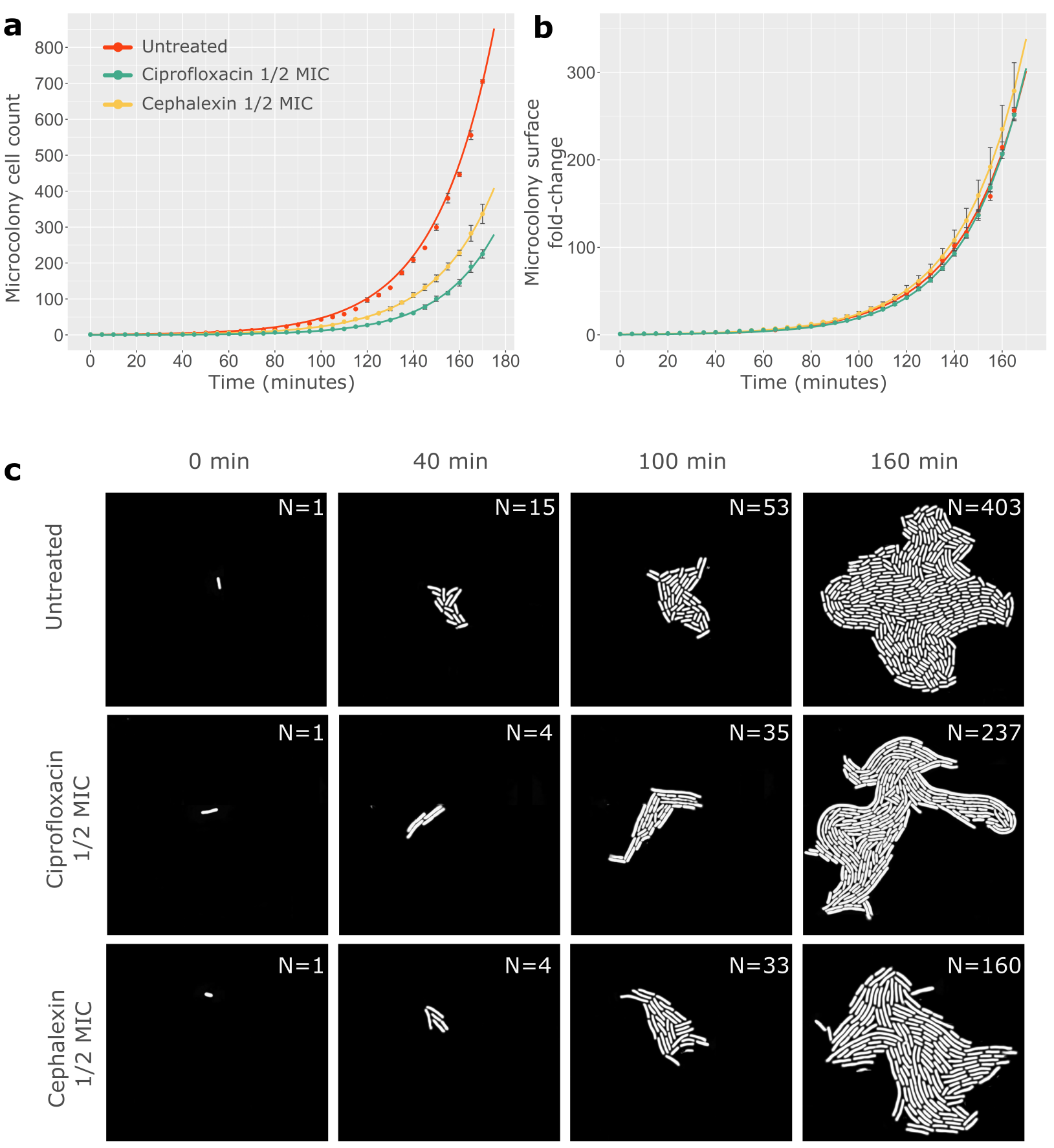

Supplement: S3 Fig — (a) Average number of fully-segmented cells in an E. coli microcolony over time at 37°C, starting from a single cell. Average generation times were of 17:50 min, 20:25 min and 21:30 min for the untreated, ciprofloxacin and cephalexin conditions respectively. (b) Average surface fold-change in the same microcolonies. (c) Binary mask of the fully segmented microcolonies obtained through MiSiC. (TIF) [file ppat.1011602.s004.tif]

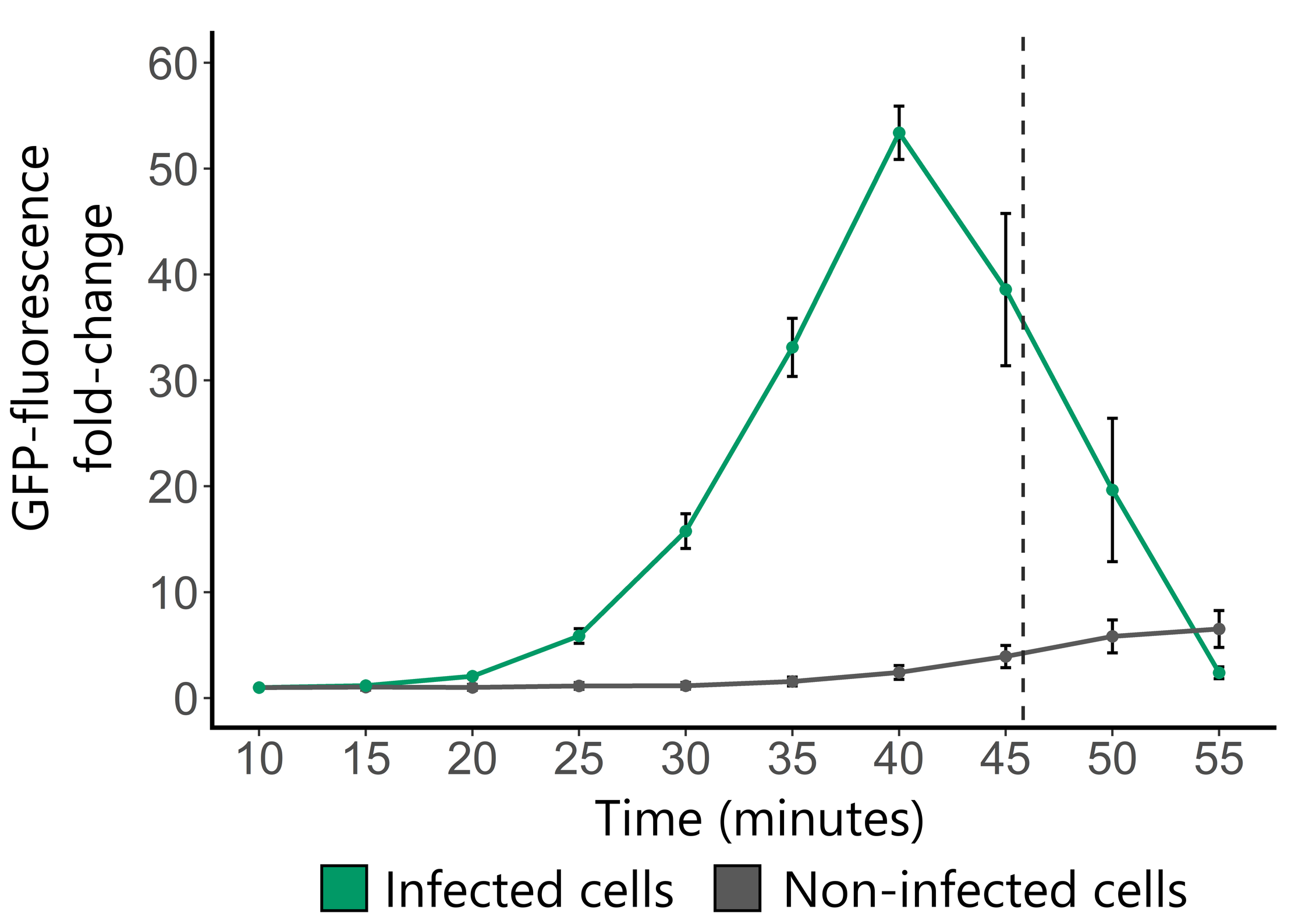

Supplement: S4 Fig — Comparison of fluorescence intensity fold-change over time between infected and uninfected cells in S1 Movie. E. coli TD2158 PL4 and phage HK620 hkcEF::PrrnB-gfp were mixed at MOI = 1 at time = 0 minutes. Intensity was measured for N > 15 bacteria belonging to each group. Vertical-dashed lines represent the lysis of the fluorescent cells. (TIF) [file ppat.1011602.s005.tif]

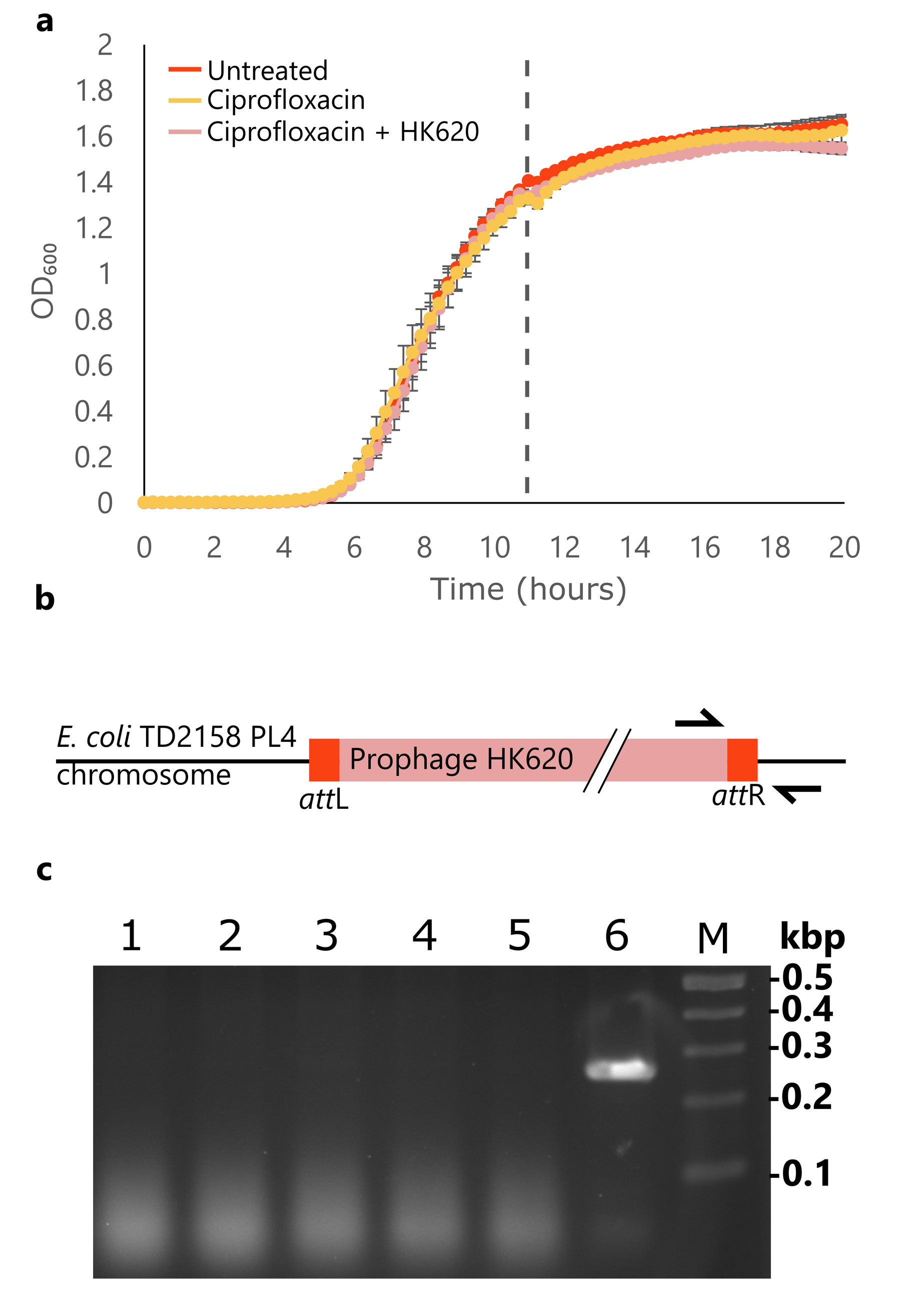

Supplement: S5 Fig — (a) TD2158 PL4 growth curves untreated, in the presence of ciprofloxacin (½ MIC), or with both ciprofloxacin (½ MIC) and phage HK620 (30 PFU/well). The dashed line represents the time of phage addition (time = 11 hours). (b) Schematic representation of primer design to screen for HK620 integration. If the prophage is present, a fragment of 264 bp will be amplified. (c) The resulting colony-PCR on pooled clones recovered after 9 hours of infection (time = 20 hours) revealing the absence of the integrated phage (lanes 1 to 5) and a positive control of a TD2158 PL4 HK620 lysogen (lane 6). M = molecular weight marker. (TIF) [file ppat.1011602.s006.tif]
