# Supplementary material for: Phage-antibiotic synergy: Cell filamentation is a key driver of successful phage predation
Source: PLoS Pathog. 2023 Sep 13;19(9):e1011602. doi: 10.1371/journal.ppat.1011602 (PMC10519598; doi:10.1371/journal.ppat.1011602)
Supplement: S1 Text — (DOCX) [file ppat.1011602.s007.docx]

# Supporting text

We model the transient dynamics of a population of bacteria subject to a combination of phage and antibiotic treatments. The density of the wild-type bacteria is noted $S_{w}$. In the absence of antibiotic these cells have a “normal size” (i.e. no filamentation) and reproduce at a density-dependant rate $r\left( 1-\frac{N}{K} \right)$ where $r$ is the maximal growth rate, $N$ is total population size of the bacteria and $K$ is the carrying capacity of the bacteria population (i.e. the maximal cell density). Bacteria are assumed to die at a constant rate $d$.

Bacteria may be exposed to a sublethal dose of antibiotic ($\sigma$ measures the rate of exposition to antibiotics) which does not kill the bacteria but induces filamentation ($F_{w}$ is the density of filamenting bacteria). Filamenting bacteria stop reproducing but we assume they can recover a regular size at rate $\gamma$. Filamenting bacteria are known to activate DNA repair systems and thus to exhibit higher mutation rates (Gutierrez et al. 2013, Bos et al. 2015). We track the accumulation of mutant bacteria that acquired resistance to other antibiotics. The variables that refer to the densities of these mutant bacteria are indicated with a subscript $m$ while we use the subscript $w$ for wild-type cells. These mutant bacteria emerge from “normal size” cells at rate $\mu_{S}$ and from filamentous cells at a higher rate $\mu_{F}$ to account for their higher mutation rate (*i.e.* $\mu_{F}>\mu_{S}$). Mutant bacteria are assumed to reproduce and die at the same rate as the wild-type cells.

Bacteria may also be exposed to a virulent phage which kills the infected cells ($V$ is the density of free viral particles). The phage life cycle starts with the adsorption of the viral particle to the $S$ and $F$ cells at rates $a_{S}$ and $a_{F}$, respectively. The lysis of $S$ and $F$ infected cells release $B_{S}$ and $B_{F}$ of new viral particles, respectively. These viral particles adsorb to new bacteria or die at a constant rate $d_{v}$. This yields the following dynamical system (see Fig. 5a):

| $\frac{dS_{w}}{dt}=r\left( 1-\mu_{S} \right)S_{w}\left( 1-\frac{N}{K} \right)+\gamma F_{w}-\left( d+a_{S} V+\sigma\right)S_{w}$  $\frac{dS_{m}}{dt}=r\left( \mu_{S}S_{w}+S_{m} \right)\left( 1-\frac{N}{K} \right)+\gamma F_{m}-\left( d+a_{S} V+\sigma\right)S_{m}$  $\frac{dF_{w}}{dt}=\sigma S_{w}-\left( d+a_{F} V+\gamma+\mu_{F} \right)F_{w}$  $\frac{dF_{m}}{dt}=\sigma S_{m}+\mu_{F}F_{w}-\left( d+a_{F} V+\gamma\right)F_{m}$  $\frac{dV}{dt}=\left( a_{S}\left( S_{w}+S_{m} \right)\left( B_{S}-1 \right)+a_{F}\left( F_{w}+F_{m} \right)\left( B_{F}-1 \right) \right)V-d_{v}V$ | (1) |
| --- | --- |

The total bacteria population size is defined as: $N=S+F$ with $S=S_{w}+S_{m}$ and $F=F_{w}+F_{m}$.

## Demography

First, we focus on the transitory effects of antibiotics and phages on the density of bacteria. In other words, we ignore the mutations and focus on the dynamics of $S$ and $F$ which yields:

| $\frac{dS}{dt}=rS\left( 1-\frac{N}{K} \right)+\gamma F-\left( d+a_{S} V+\sigma\right)S$  $\frac{dF}{dt}=\sigma S-\left( d+a_{F} V+\gamma\right)F$  $\frac{dN}{dt}=rS\left( 1-\frac{N}{K} \right)-d N- V\left( a_{S}S+a_{F}F \right)$ | (2) |
| --- | --- |

The final term of the last equation captures the effect of viruses on the bacterial population (see Fig. 1c). We can use our model to simulate the change in the biomass $S+\frac{a_{F}}{a_{S}}F$ of cells which accounts for the increased size of the filamenting bacteria which is also responsible for the increases adsorption rate of the phage. This yields Figure 5b which captures the synergy between antibiotics and phages (compare Fig. 5b and Fig. 1c).

This effect depends on the densities of $S$ and $F$ cells. The dynamics of the frequency $f_{F}=\frac{F}{N}$ of filamentous cells is given by:

| $\frac{df_{F}}{dt}=\underset{antibiotic}{\underbrace{\sigma\left( 1-f_{F} \right)}}-\underset{recovery}{\underbrace{\gamma f_{F}}}-\underset{no reproduction}{\underbrace{rf_{F}\left( 1-f_{F} \right)\left( 1-\frac{N}{K} \right)}}- \underset{bacteriophage}{\underbrace{Vf_{F}\left( 1-f_{F} \right)\left( a_{F}-a_{S} \right)}}$ | (3) |
| --- | --- |

This equation captures the effect of antibiotics on the proportion of filamentous cells (i.e. higher concentration of antibiotics increases the proportion of filamentous cells). This equation also shows that a higher density of viruses can reduce the proportion of filamentous cells when $a_{F}>a_{S}$. The reduction of the proportion of filamentous cells when cultures are exposed to bacteriophages (see Fig. 4) indicates that $a_{F}>a_{S}$.

## Evolution

Second, we focus on another indirect benefit of combination therapy emerging from the reduction of the influx of mutations in the bacteria population. To better understand the transient evolutionary dynamics of resistance we focus on dynamics of the frequency of antibiotic resistance $f_{m}=\frac{S_{m}+F_{m}}{N}$ which yields:

| $\frac{df_{m}}{dt}=r\left( 1-\frac{N}{K} \right)\underset{\begin{aligned} mutation \\ from S \end{aligned}}{\underbrace{\mu_{S}f_{w}^{S}f_{S}}}+\underset{\begin{aligned} mutation \\ from F \end{aligned}}{\underbrace{\mu_{F}f_{w}^{F}f_{F}}}+\underset{growth}{\underbrace{r\left( 1-\frac{N}{K} \right)\left( f_{m}^{S}-f_{m} \right)f_{S}}}+\underset{bacteriophage}{\underbrace{Vf_{F}\left( a_{F}-a_{S} \right)\left( f_{m}-f_{m}^{F} \right)}}$ | (4) |
| --- | --- |

where: $f_{m}^{S}=\frac{S_{m}}{S}$, $f_{m}^{F}=\frac{F_{m}}{F}$, $f_{w}^{S}=\frac{S_{w}}{S}$, $f_{w}^{F}=\frac{F_{w}}{F}$.

The first two terms in equation (4) account for the effects of mutation rates. In particular, the second term captures the increased mutation rate in filamentous cells. The final two terms depend on the distribution of the mutant between the $S$ and the $F$ cells.

To analyse the build-up of this distribution of mutants we can track the frequency of mutations in the two compartments which yields:

| $\frac{df_{m}^{S}}{dt}=r\left( \mu_{S}\left( 1-f_{m}^{S} \right) \right)\left( 1-\frac{N}{K} \right)+\left( f_{m}^{F}-f_{m}^{S} \right)\gamma\frac{F}{S}$  $\frac{df_{m}^{F}}{dt}=\mu_{F}\left( 1-f_{m}^{F} \right)+\sigma\frac{S}{F}\left( f_{m}^{S}-f_{m}^{F} \right)$ | (5) |
| --- | --- |

Let us define $\Delta=\left( f_{m}^{F}-f_{m}^{S} \right)$:

$$\frac{d\Delta}{dt}=\mu_{F}\left( 1-f_{m}^{F} \right)-r\left( \mu_{S}\left( 1-f_{m}^{S} \right) \right)\left( 1-\frac{N}{K} \right)-\Delta\left( \sigma\frac{S}{F}+\gamma\frac{F}{S} \right)$$

The dynamics of the above equation is driven by the first term when $\mu_{F}\gg\mu_{S}$ and implies that $\Delta>0$ and thus that $f_{m}^{F}>f_{m}^{S}$. Indeed, the frequency of the mutant increases in the $F$ cells before diffusing in the $S$ cells. This means that both the third and the fourth terms in (4) are negative and tend to slow down the increase in $f_{m}$ induced by the high mutation rate $\mu_{F}$ in $F$ cells.

Increasing phage density acts on the change in mean frequency via the fourth term in (4) (higher phage density decreases the fourth term) but it also acts via the frequency of $F$ cells (see equation (3)). Decreasing the density of $F$ cells reduces the influx of new mutations and reduces dramatically the frequency of mutations. We illustrate the effect of phages on antibiotic-induced bacterial mutagenesis in Figure 5c.
